# Extreme anoxia tolerance in crucian carp and goldfish through neofunctionalization of duplicated genes creating a new ethanol-producing pyruvate decarboxylase pathway

**DOI:** 10.1038/s41598-017-07385-4

**Published:** 2017-08-11

**Authors:** Cathrine E. Fagernes, Kåre-Olav Stensløkken, Åsmund K. Røhr, Michael Berenbrink, Stian Ellefsen, Göran E. Nilsson

**Affiliations:** 1 Department of Biosciences, University of Oslo, N-0316 Oslo, Norway; 2 Institute of Basic Medical Sciences, University of Oslo, N-0372 Oslo, Norway; 3 Center for Heart Failure Research, University of Oslo, N-0317 Oslo, Norway; 4 Institute of Integrative Biology, University of Liverpool, Liverpool, L69 7ZB United Kingdom; 5 The Lillehammer Research Center for Medicine and Exercise Physiology, Inland Norway University of Applied Sciences, N-2604 Lillehammer, Norway

## Abstract

Without oxygen, most vertebrates die within minutes as they cannot meet cellular energy demands with anaerobic metabolism. However, fish of the genus *Carassius* (crucian carp and goldfish) have evolved a specialized metabolic system that allows them to survive prolonged periods without oxygen by producing ethanol as their metabolic end-product. Here we show that this has been made possible by the evolution of a pyruvate decarboxylase, analogous to that in brewer’s yeast and the first described in vertebrates, in addition to a specialized alcohol dehydrogenase. Whole-genome duplication events have provided additional gene copies of the pyruvate dehydrogenase multienzyme complex that have evolved into a pyruvate decarboxylase, while other copies retained the essential function of the parent enzymes. We reveal the key molecular substitution in duplicated pyruvate dehydrogenase genes that underpins one of the most extreme hypoxic survival strategies among vertebrates and that is highly deleterious in humans.

## Introduction

When a vertebrate is deprived of oxygen, an inability to match ATP supply and demand generally leads to death within minutes^1^. However, a few vertebrates have evolved mechanisms to handle anoxia, allowing them to overwinter in oxygen deprived ice-covered ponds and shallow lakes^2, 3^. These include cyprinid fishes of the genus *Carassius*, the crucian carp (*C. carassius*) and the goldfish (*C. auratus*), which display the most extreme anoxia tolerance among teleosts, surviving without oxygen for up to 4–5 months, limited only by the exhaustion of large liver glycogen stores^4–6^. The pioneering work by Shoubridge and Hochachka revealed that the biochemical adaptations to anoxia in *Carassius* depend on the ability of their skeletal muscles to convert anaerobically produced lactic acid into ethanol, which can freely diffuse across the gills into the ambient water, thereby avoiding lactic acidosis^7–9^. The importance of this ethanol pathway is underlined by observations made in the closely related common carp *(Cyprinus carpio*), which tolerates severe hypoxia^10^ but dies within hours of anoxia, apparently related to accumulation of lactate due to its inability to produce ethanol^11^.

Ethanol formation in *Carassius* skeletal muscle was early attributed to a pyruvate dehydrogenase complex (PDHc) supposedly releasing acetaldehyde, and an alcohol dehydrogenase (ADH) converting acetaldehyde to ethanol^9, 12^.

PDHc is the largest known multienzyme complex among eukaryotes (mass ~9 MDa)^13, 14^, providing the essential link between the glycolytic pathway and the tricarboxylic acid (TCA) cycle^14^. It consists of multiple copies of three catalytic components; Enzyme 1 (E1, pyruvate dehydrogenase; a 2α2β tetramer), Enzyme 2 (E2, dihydrolipoamide transacetylase) and Enzyme 3 (E3, dihydrolipoamide dehydrogenase, a homodimer)^14–17^. These enzymes work in a sequential manner, catalysing the conversion of pyruvate into acetyl-CoA through substrate channelling and a number of intermediate steps including the transformation of pyruvate into CO_2_ plus an acetyl group by E1^14^. In the absence of oxygen, acetyl-CoA cannot be further metabolized as the TCA cycle is at a halt, and it has previously been speculated that *Carassius* PDHc becomes “leaky” or even partly dissociates in anoxia allowing this acetyl group to be released as acetaldehyde^9, 18^. However, despite detailed structure-function and molecular evolutionary analyses of these enzyme systems, and their importance in several human disease contexts^19–21^, the origin and molecular basis of the metabolic pathway that allows extreme anoxia tolerance in selected vertebrates has remained unknown.

## Results and Discussion

Here we provide evidence to suggest that in addition to a normal PDHc, *Carassius* possess an alternative E1 enzyme that is activated during anoxia and functions as an acetaldehyde-producing mitochondrial pyruvate decarboxylase (PDC) analogous to the cytosolic pyruvate decarboxylase in brewer’s yeast (see ref. 18). This is made possible through the presence in *Carassius* of an additional set of paralogs of each of the E1α and E1β subunits. While one pair maintained the original function (i.e. catalysing synthesis of acetyl-CoA during normoxia as an integral part of PDHc), the other pair has apparently evolved into an E1 enzyme physically independent of PDHc, catalysing the formation of acetaldehyde in anoxia, which can then be effectively converted into ethanol by a muscle-specific ADH^7–9, 11^.

To unravel the molecular basis and origin of vertebrate ethanol production and thus anoxia tolerance, we have identified multiple PDHc and ADH transcripts from tissues of anoxia tolerant and intolerant cyprinid species. We then compared the tissue specific expression patterns of these transcripts in crucian carp after normoxic exposure (7 days, N7), 1 or 7 days of anoxia (A1, A7), and reoxygenation (R, for 6 days after 7 days of anoxia), using an external RNA reference for normalization^22^.

**Figure 1:**
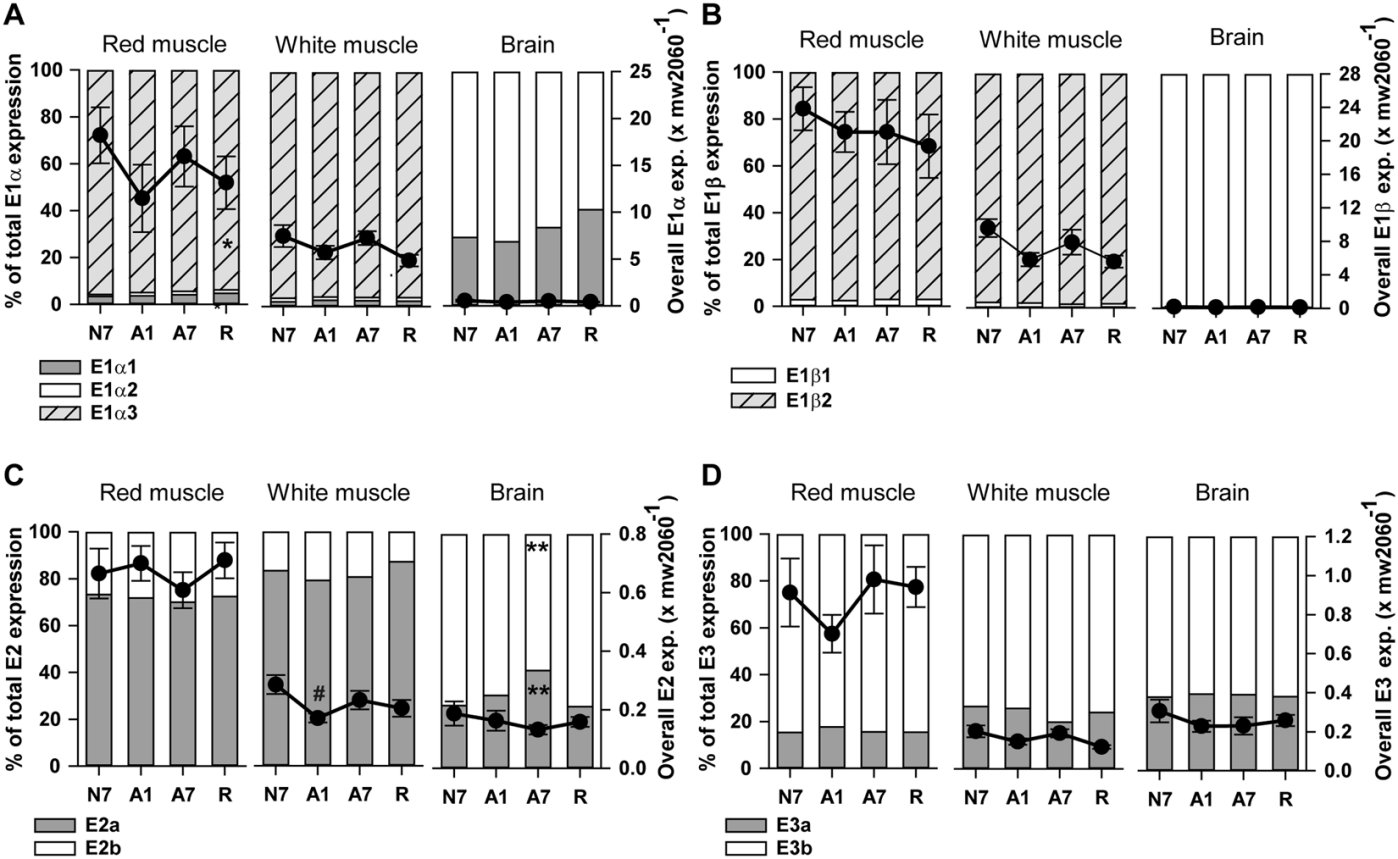
mRNA transcript levels of PDHc subunits (**A**) E1α, (**B**) E1β, (**C**) E2 and (**D**) E3 in red muscle, white muscle and brain of crucian carp. X axes show treatment groups: normoxic control (N7), 1 day anoxia (A1), 7 days anoxia (A7) and reoxygenation (R). Left y-axes and bars show percentage distribution of paralogs, while right y-axes and filled circles show overall expression levels of genes (means ± S.E.M. of all paralogs combined; n = 6–8 fish per group). Significant difference compared to N7 is indicated by * for percentage distribution data and # for overall expression. */^#^P > 0.05; **/^##^P > 0.01; ***/^###^P > 0.001 (One-way ANOVA; Holm-Sidak post-hoc test).

**Figure 2:**
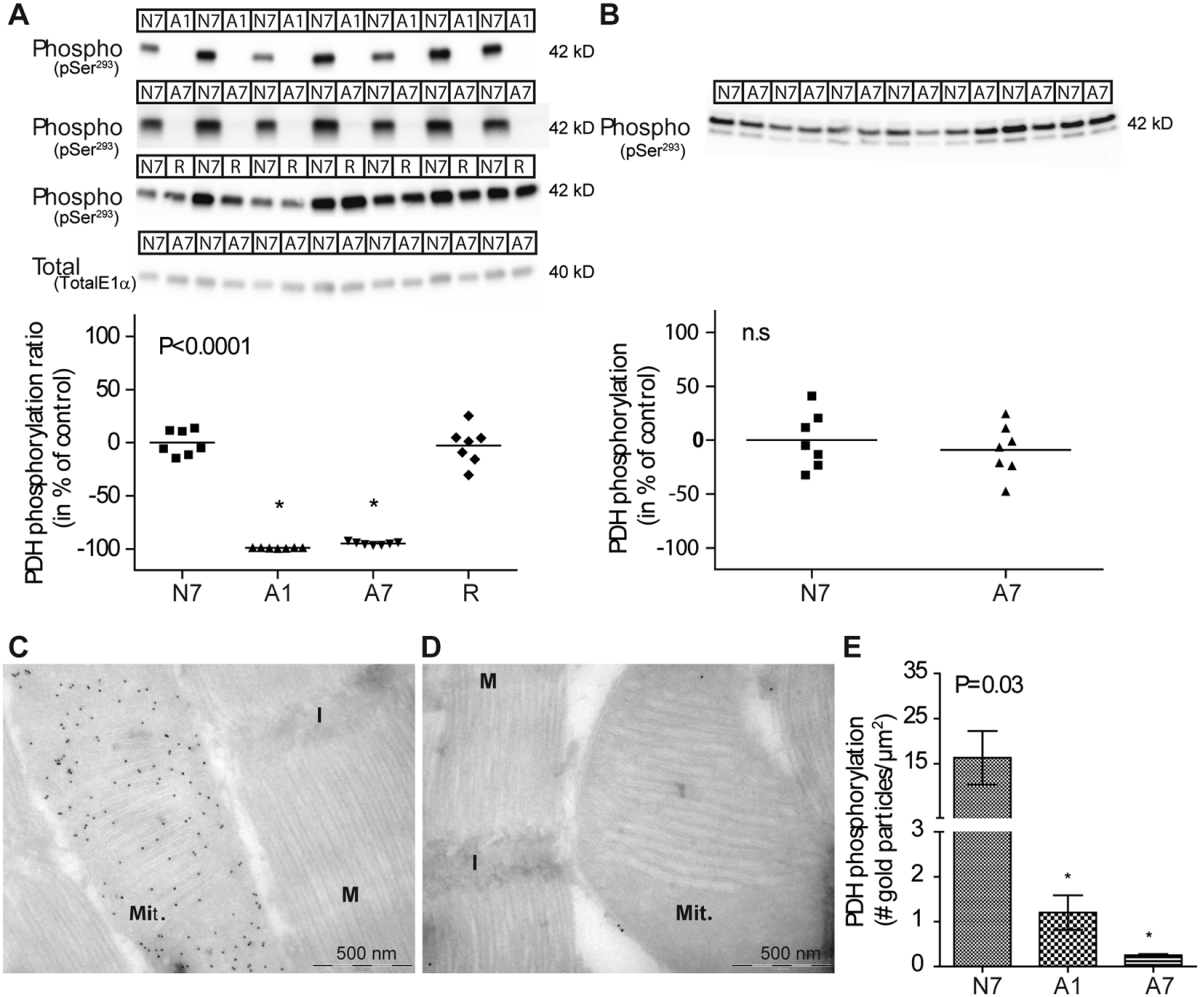
Phosphorylation of crucian carp E1α subunits in normoxia (N7) and anoxia (A7). (**A**,**B**) Phosphorylation of E1α subunits in relation to N7 (n = 7) are shown for red muscle (**A**) and brain (**B**). Western blot images include staining using antibodies against phosphorylated PDHc (upper three images in **A** and image in **B**) and against total E1α (lower image in **A**). Statistical difference compared to N7 is indicated by * (p < 0.0001). n.s = not significant; kD = kilodalton. **(C**,**D)** EM micrographs of crucian carp red muscle showing gold-staining of phosphorylated E1α subunits during normoxia **(C)** and anoxia **(D)**. Mit. = mitochondria; I/M = visible bands of muscle fibers. **(E)** Quantification of gold particles per µm^2^ in red muscle following N7, A1 and A7 (Means ± S.E.M. of 3 individuals per group). (See legend to Fig. 1).

Anoxia exposure of crucian carp for as long as 7 days had only negligible effects on PDHc subunit transcript levels across all examined tissues and paralogs. (Fig. 1, Supplementary Figs S1–S3; see also Supplementary Table S4 for a full list of transcripts), suggesting a constitutive ability for anoxic ethanol production in *Carassius*, in line with its ability to tolerate acute insults of anoxia^8^. However, PDHc subunit transcription was highly tissue specific, with E1α_3_, E1β_2_, and E2a transcripts dominating in ethanol-producing red and white skeletal muscle, and E1α_1_ or E1α_2_, E1β_1_, and E2b transcripts dominating in brain, liver, and heart (Fig. 1; Supplementary Figs S1–S3). The analysis further revealed a suspiciously large excess of E1 transcripts in *Carassius* red and white muscle, wherein overall E1α and E1β mRNA levels were one to two orders of magnitude higher than in brain, heart and liver (Fig. 1A,B; Supplementary Figs S1 and S3; Supplementary Table S5). The large excess of E1 transcript levels in skeletal muscle was reflected in very high E1α protein levels compared to brain in the Western blot analyses (Fig. 2A,B; see also Methods and Supplementary Fig. S6 for more details). In contrast, in anoxia intolerant common carp, red muscle and brain yielded similar levels of overall E1α and E1β mRNA transcripts, which corresponded to levels in *Carassius* brain and were very much below levels in *Carassius* skeletal muscle (Supplementary Fig. S3).

The excess E1 transcript level in crucian carp red muscle was entirely due to a pair of specific E1α and E1β paralogs, here denoted E1α_3_ and E1β_2_, which dominated the overall E1α and E1β transcript levels with 95.7 ± 0.4% and 97.1 ± 0.4% of the total (Fig. 1A,B). A similar picture emerged in crucian carp white muscle (Fig. 1A,B) and in goldfish red muscle (Supplementary Fig.S3). By contrast, transcript levels of these paralogs were minimal or non-detectable in *Carassius* brain, liver and heart (Fig. 1A,B; Supplementary Figs S1 and S3).

Molecular phylogenetic analyses suggest that the dominating E1α_3_ and E1β_2_ paralog pair in *Carassius* skeletal muscles originates from a cyprinid-specific paleotetraploidization event that occurred approximately 8.2 million years ago (MYA) in a common ancestor of the *Carassius* species and the common carp^23^ (Supplementary Fig. S7). Indeed, the common carp also has an extra set of E1α and E1β paralogs, although their transcripts present a minimal fraction of the other paralogs and are expressed at negligible levels in both brain and red muscle in this species. Analysis of publicly available sequences of zebrafish *(Danio rerio)*, which diverged from the above cyprinids prior to this tetraploidization event, only revealed one E1β paralog and two E1α paralogs that originated from a basal split of teleost E1α paralogs into an E1α_1_/E1α_3_ and an E1α_2_ clade, consistent with their origin from the ancient teleost genome duplication event some 100–200 MYA^24^.

Together, these data suggest that the protein products of the E1α_3_ and E1β_2_ paralogs combine to produce a specific E1 enzyme that is expressed at levels far in excess of what is needed for PDHc and that is responsible for the decarboxylation of pyruvate into acetaldehyde in *Carassius* skeletal muscle, i.e. having become a PDC. This comprises the first description of a PDC in any vertebrate, representing a type of enzyme that is otherwise best known from ethanol-producing organisms such as yeast^25^ and plants^18^.

To ensure normal aerobic PDHc functions in *Carassius* tissues, i.e. to allow for production of acetyl-CoA and NADH, all tissues including skeletal muscle need to express complete sets of the PDHc subunits E1α_1–2_ and E1β_1_, E2a-b, E3a-b, and the structural component E3-binding protein (E3BP)^16, 17^, and need to do so in proper stoichiometric relationships. In line with this, comparable levels of mRNA transcripts of these PDHc components were found in all examined crucian carp tissues (Fig. 1; Supplementary Figs S1–S3), with the slightly elevated mRNA transcript levels in red skeletal muscle (Fig. 2; Supplementary Fig. S1) consistent with the higher mitochondrial content of this tissue^26^. mRNA expression level ratios between total E1, E2 and E3 paralog transcripts were largely consistent between brain, heart and liver tissues, and the E2: E3 transcript ratio varied within narrow margins across all examined tissues, with no significant differences between the brain and the red and white skeletal muscles (Supplementary Table S5). In sharp contrast to this, in red and white skeletal muscles, total E1:E2 and E1:E3 transcript ratios were significantly higher by a factor greater than 60 compared to all other, non-ethanol producing tissues. (Supplementary Table S5).

**Figure 3:**
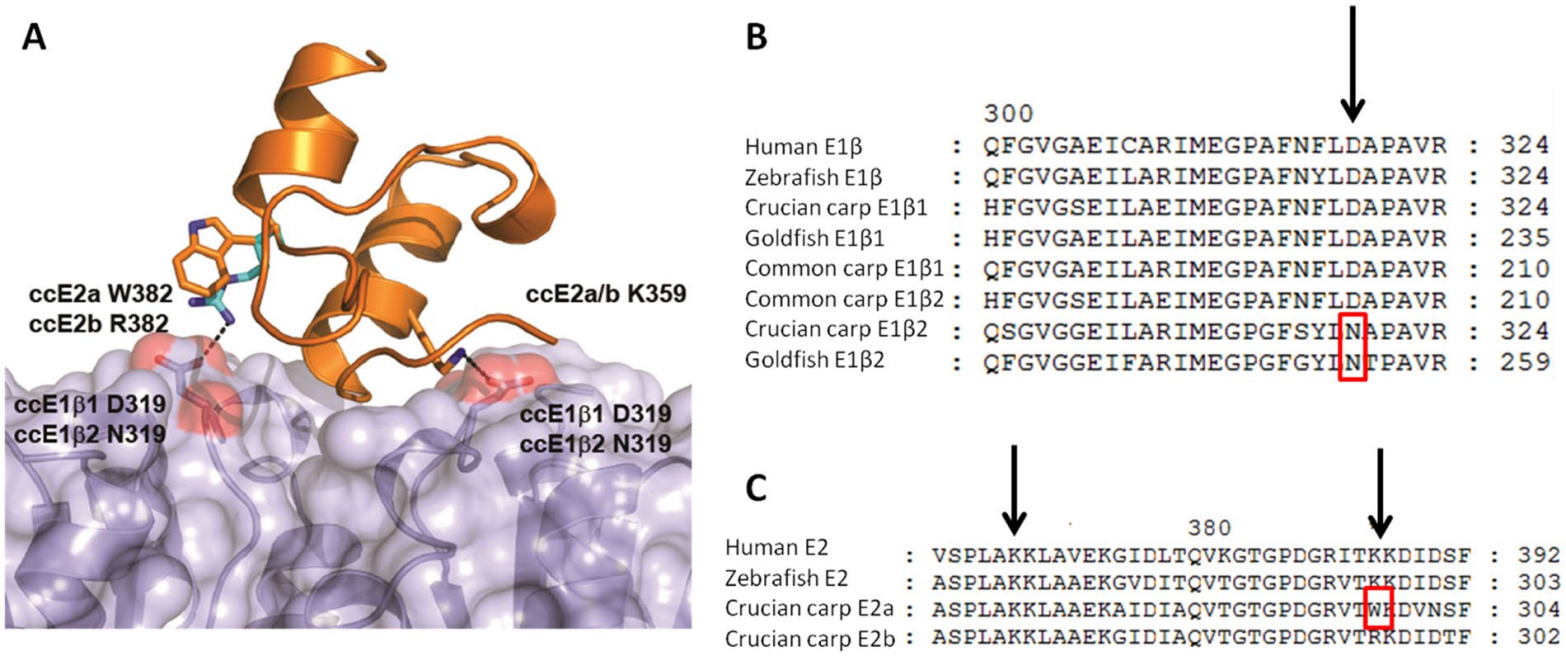
E1–E2 contact sites. **(A)** Structural model of the interaction sites between the E1β_1_ dimer (blue) and the peripheral subunit binding domain of the E2b monomer in crucian carp (cc) PDHc (orange). The highlighted E1–E2 interaction is critically dependent on the salt bridge forming ccE1β_1_ D319, which is substituted by neutral N in ccE1β_2_. Whether one or both of the indicated salt bridges between E1β dimers and E2 monomers are lost depends on which E1β and E2 paralogs that form the complex, see text for further information. **(B)** Alignment of amino acids 298–324 of E1β and **(C)** amino acids 357–392 of E2 in selected organisms (positional numbering follows the human sequences). Arrows pinpoint E1–E2 contact sites, with substitutions impairing contact boxed.

In order to allow functional differentiation without compromising the essential PDHc function, an E1-derived PDC likely needs to be prevented from attaching to E2, allowing it to operate without interference of any E2 subunit. Intriguingly, *Carassius* E1β_2_, but not E1β_1_, exhibits the amino acid substitution βD319N that has been associated with PDHc dysfunction in human cells, resulting in a >100-fold increase in the dissociation constant (K_D_) between E1 and E2 and a concomitant decrease in *in vitro* PDHc activity and functionality^27^. Molecular modelling shows that the βD319N substitution in *Carassius* E1β_2_ abolishes a crucial salt bridge at the E1–E2 binding site between βD319 and the corresponding residues K362 on E2a and K362 and K386 on E2b (Fig. 3), further strengthening our hypothesis of a separate PDC-like E1 in *Carassius*, and contrasting with earlier suggestions of a modified PDHc that leaks acetaldehyde during anoxia or even dissociation of the E1 component from the complex under anoxic conditions^18^. Importantly, this substitution is apparently unique to ethanol producing goldfish and crucian carp and not found in any other species investigated. Molecular modelling of *Carassius* E1α and E1β paralogs did not reveal additional amino acid substitutions that would be expected to alter structural or kinetic properties of E1.

Interestingly, closer examination of the skeletal muscle paralogs apparently constituting the E1–E2 contact site of the housekeeping PDHc complex revealed that the dominant skeletal muscle E2 paralog of *Carassius* shows the unique substitution of positively charged K or R for neutral W at position 382, which will abolish one of the two E1–E2 binding sites and may thus weaken the binding between E1 and E2 (Fig. 3A,C). This could further contribute to allowing the novel E1 paralogs to detach from E2, and may have been a first step in the evolution of an independent E1 functioning as a PDC. Molecular phylogenetic analysis suggests that the E2a and b paralogs originated during the same cyprinid paleotetraploidization event that also gave rise to the muscle specific overexpressed E1α and β paralogs in *Carassius*, (Supplementary Fig. S7).

The enzyme activity of *Carassius* PDC should be tightly regulated in order to balance metabolism in response to oxygen availability, likely through controlling phosphorylation/dephosphorylation of the E1α subunit^28^. Accordingly, we found E1α in crucian carp red muscle to be completely dephosphorylated and thus activated during anoxia (Fig. 2A), which was not observed in brain (Fig. 2B), where E1α phosphorylation status was unaffected. In muscle, reoxygenation led to massive phosphorylation of E1α (Fig. 2A). Notably, in crucian carp red muscle, total levels of E1α protein were not affected by anoxia (Fig. 2A), in accordance with the lack of changes seen in E1α mRNA. In brain (Fig. 2B), the observation of two bands is consistent with the finding of two paralogous mRNA transcripts in the brain (E1α_1–2_). Further studies will be needed to clarify if specific forms of kinases and phosphatases are responsible for a tissue and isoform specific differential regulation of E1 activity during normoxia and anoxia.

The presence of 5′ mitochondrial import signals in both *Carassius* PDC subunits supports a mitochondrial localization (see Supplementary Table S4 for accession numbers). In agreement with this, electron microscopy visualizing antibodies against phosphorylated E1α in red muscle revealed a mitochondrial localization, and like with Western blot, the signal disappeared in anoxia suggesting activation by dephosphorylation (Fig. 2C–E). This agrees with previous research showing that acetaldehyde is produced and released from isolated anoxic mitochondria from *Carassius* muscle^9^.

**Figure 4:**
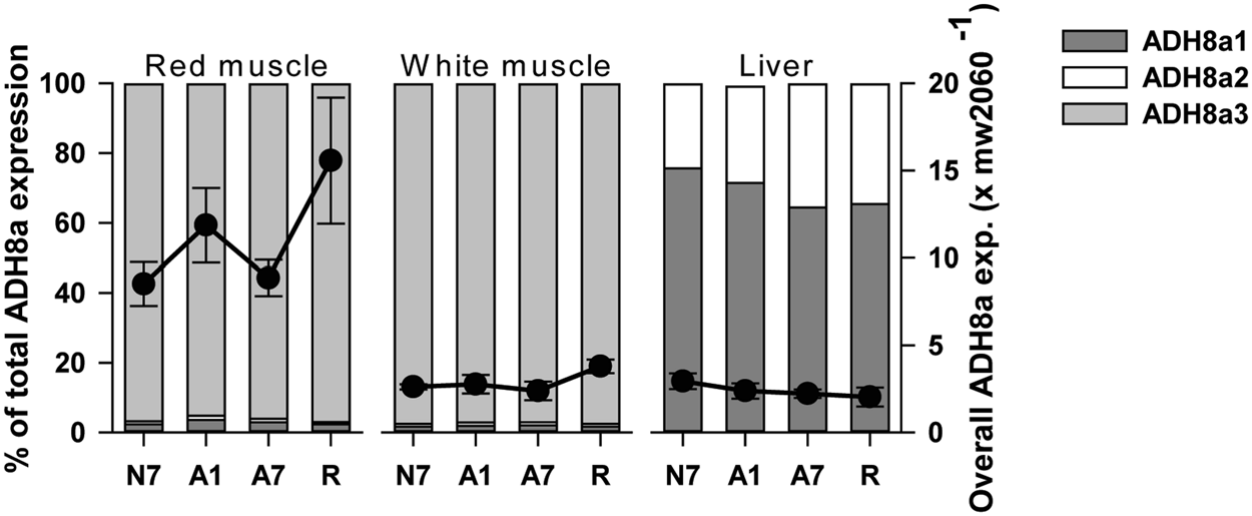
ADH8a transcript levels in red muscle, white muscle and liver of crucian carp. mRNA expression levels of ADH8a1–3 normalized to expression of mw2060 in red muscle, white muscle and liver of crucian carp. X axes show treatment groups (N7, A1, A7 and R). Left y-axes and bars show percentage distribution of paralogs, while right y-axes and filled circles show overall expression levels of genes (means ± S.E.M. of all paralogs combined; n = 6–8 fish per group). No statistical differences between groups compared to N7 were found (One-way ANOVA).

In addition to the hitherto described PDC, the ethanol-producing pathway in *Carassius* muscle also includes an ADH, more precisely ADH8a, the isoform known to interconvert acetaldehyde and ethanol in zebrafish^29^. It is normally restricted to vertebrate livers^7, 12, 30^, but we expected that a specific ADH8a paralog may be present in *Carassius* muscle and thus be responsible for reducing acetaldehyde produced by PDC to ethanol. In agreement with this, we found three ADH8a paralogs in crucian carp (denoted ADH8a1–3), possibly resulting from the previously mentioned gene duplication events^23, 31–33^ (Supplementary Fig. S8; see Supplementary Table S4 for accession numbers). Whereas the ADH8a3 mRNA completely dominated the expression in skeletal muscle, constituting 96% of total ADH8a mRNA, it was absent in liver (Fig. 4), where ADH8a1 dominated. The overall ADH8a mRNA level in red muscle was higher by a factor of 4 compared to white muscle and liver (Fig. 4), corresponding well to the reported higher maximal enzyme reaction velocity V_max_ of ADH in red muscle compared to white muscle by a factor of 3.6^12^. Comparison of the *Carassius* ADH8a3 sequence with the orthologous Baltic cod *(Gadus morhua callarias)* sequence revealed a Pro to His substitution at position 122 that is located in the tunnel leading into the active site, potentially affecting substrate channelling (Supplementary Fig. S9). This could be related to the high affinity for acetaldehyde and low affinity for ethanol displayed by ADH in *Carassius* muscle^9^, although the functional importance of this substitution for ethanol production cannot be elucidated further from this model.

**Figure 5:**
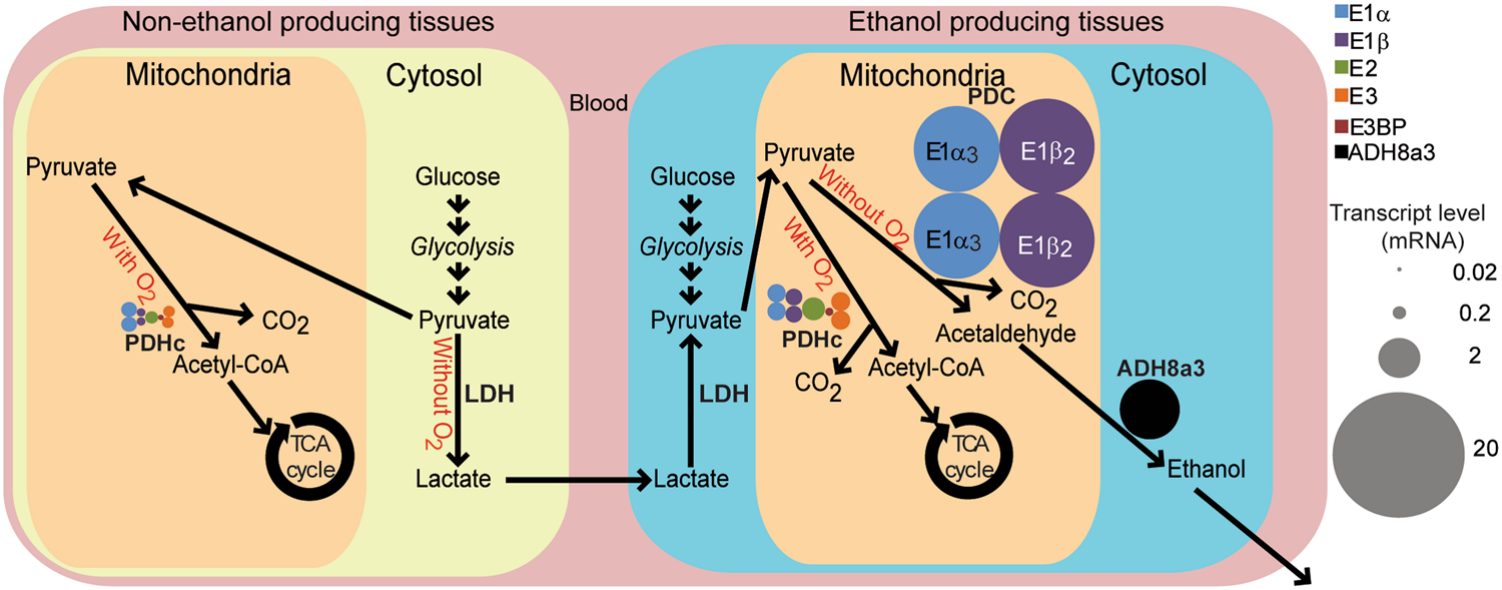
Model for the alternative routes of pyruvate metabolism in *Carassius*. Model for pyruvate handling in *Carassius* non-ethanol producing tissues (left), contrasted with pyruvate handling in ethanol producing tissues (right). Combined areas of similarly colored circles correspond to mRNA levels in brain (left) and red skeletal muscle (right) from crucian carp. In case of dimers, total transcript levels were halved, and represented as dimers in the figure, together representing the total transcript level of the corresponding gene. Bubble size is scaled according to mRNA transcript level. In non-ethanol producing tissues, pyruvate from glycolysis is either converted to acetyl-CoA and CO_2_ (with oxygen) by the pyruvate dehydrogenase complex (PDHc) or to lactate by lactate dehydrogenase (LDH; without oxygen), as evident from the lack of expressed PDC components. In ethanol producing tissues, pyruvate from glycolysis is either processed to acetyl-CoA and CO_2_ by PDHc for further metabolism in the TCA cycle when oxygen is present, or converted into ethanol through the PDC-ADH pathway during anoxia.

This study has identified and characterized components of a novel, muscle-specific ethanol-producing metabolic pathway in the teleost genus *Carassius* that enables extreme anoxia tolerance^7, 8^, while its tetraploid genome has allowed the traditional PDHc function to be retained. The ethanol-producing and the acetyl-CoA producing pathways are summarized in Fig. 5. This novel PDC-ADH pathway has likely evolved by means of a cyprinid-specific genome size duplication that provided the genomic framework of paralogs for spatial and functional differentiation. Completion of the recently published draft genome of the paleotetraploid common carp^23^ holds great promise to further elucidate the molecular basis and origin of the ethanol pathway in the genus *Carassius*, by providing a complete set of paralogs of all relevant components their synteny and chromosomal location.

Cyprinids like the goldfish have a long history of use in ethanol toxicity studies^34^ and zebrafish are emerging model systems for the study of alcohol tolerance and sensitization, and foetal alcohol syndrome^35, 36^. Members of the *Carassius* lineage are naturally exposed to elevated tissue ethanol concentrations reaching up to 7 µmol/g in red muscle during anoxia for up to 4–5 months of the year^8^, and we suggest that they also hold promise as a model systems for the study of molecular mechanisms protecting against chronic ethanol exposure.

Finally, the evolution of the ethanol-producing pathway has not only made the goldfish one of the arguably most resilient pets under human care, but has also clearly provided *Carassius* with unique ecological benefits, allowing survival in waters that are uninhabitable for other fish, thereby evading piscine predation and interspecific competition.

## Methods

### Animals

Crucian carp of mixed sex were caught in a small pond outside of Oslo, Norway, and kept in 750-litre tanks continuously supplied with dechlorinated, aerated Oslo tap water in the aquarium facilities at the Department of Biosciences, University of Oslo, Norway. The photoperiod was kept constant at 12 h/12 h intervals of light/darkness, and water temperature was around 6 °C. The fish were fed commercial carp food (Tetrapond, Tetra, Melle, Germany) on a daily basis. These fish were later subjected to anoxia exposures, as described below. Goldfish and common carp (obtained commercially) were transported to the aquarium facilities at the University of Oslo, where they were sacrificed at the day of arrival.

### Anoxia exposures and tissue sampling

Prior to anoxia exposure experiments, randomly selected crucian carp were transferred to experimental tanks with continuous flow-through water supply, and left to acclimate and fast for at least 30 h. Subsequently, these tanks were sealed with tight lids allowing no light to shed through, and the water was continuously bubbled with nitrogen gas (anoxia; AGA) or regular air (normoxia and reoxygenation) through a narrow and 2 m high Plexiglas column to equilibrate the gas with the water. Throughout the experimental period, temperature and oxygen saturation in the tanks were continuously monitored using a galvanometric oxygen electrode (Oxi 340i; WTW, Weilheim, Germany). Water with no detectable oxygen (<0.01 mg O_2_ l^−1^), well below the anaerobic threshold of crucian carp (0.5 mg O_2_ l^−1^)^37^, was considered anoxic. Upon termination of the exposures, all fish were killed by stunning them with a sharp blow to the head, followed by rapid cutting of the spinal cord. All animal experiments were performed with approval from The National Animal Research Authority of Norway (permit nr 12007), and all methods involving research animals were performed in accordance with relevant guidelines and regulations. No death was observed during the experiment.

#### Anoxia exposure series

*In anoxia exposure series I*, randomly selected crucian carp (49 ± 2 g) were divided into four experimental groups (n = 13/group): 7 days of normoxia (N7), 1 day of anoxia (A1), 7 days of anoxia (A7) and reoxygenation (7 days of anoxia followed by 6 days of normoxia; R), and kept in 25 l tanks. Anoxic exposures in this series were conducted at 6.5 ± 0.3 °C during January on fish caught during the previous summer. Tissues were immediately dissected in the following order: (a) brain (b) heart (c) liver, (d) red skeletal muscle and (e) white skeletal muscle, and snap-frozen in liquid nitrogen within 2 min of the initial handling. Tissues were stored at -80 °C until further use in cloning, qPCR or Western blotting experiments.

*Anoxia exposure series II:* In anoxia exposure series II, randomly selected crucian carp (16 ± 1 g) were divided into three experimental groups (n = 3/group): 7 days of normoxia at 4 °C (N7), 1 day of anoxia at 4 °C (A1) and 7 days of anoxia at 4 °C (A7). Fish acclimated at 4 °C were kept in a 15 l tank with eight compartments of 1.5 l each that were housed in a cold room. Immediately after death, a red skeletal muscle strip (20 mm) from each individual was dissected free from the white muscle and transferred to a chamber containing 0.1% glutaraldehyde and 4% paraformaldehyde in phosphate-buffered saline (PBS). The length of the muscle was maintained by two insect pins during the fixation process. After two hours, a 1 mm^3^ block was cut (with one side containing the skin) and transferred to a tube containing the same fixatives and stored at 4 °C for later use in electron microscopy studies.

### Obtaining sequences for the pyruvate dehydrogenase complex and alcohol dehydrogenase

To our knowledge, none of the genetic components of the pyruvate dehydrogenase complex or alcohol dehydrogenase in the *Carassius* lineage had been characterized prior to these experiments. Consequently, cloning and sequencing were demanded in order to design species-specific primers for quantitative real-time RT-PCR (qPCR) for *Carassius* and common carp. Primers were designed from sequences of zebrafish *(Danio rerio)*, belonging to the same family as *Carassius* (Cyprinidae), using Primer3^38^, and were synthesized by Invitrogen (Invitrogen, Carlsbad, CA, USA). The GSPs are listed in Supplementary Table S4. The primers were designed in regions displaying high degree of conservation between zebrafish and other vertebrates, for which sequences were retrieved from the National Center for Biotechnology Information (NCBI; www.ncbi.nlm.nih.gov/) and Ensembl Genome Browser (www.ensembl.org/index.html) databases. Sequences were aligned using GeneDoc (version 2.7; http://www.psc.edu/biomed/genedoc) and ClustalX version 2.0.12^39^.

For cloning, total RNA was extracted from normoxic tissues of *Carassius* and common carp using TRIzol reagent (Invitrogen, Carlsbad, CA, USA) and an electric homogenizer (Ultra-Turrax T8, IKA, Staufen, Germany). Quality and quantity of the total RNA were assessed using a 2100 BioAnalyzer with RNA 6000 Nano Lab Chip Kit (Agilent Technologies, Palo Alto, CA, USA) and NanoDrop 2000 UV-Vis Spectrophotometer (Thermo Fisher Scientific, Rockland, DE, USA). All samples passed these validity checks. All procedures were carried out according to the manufacturer’s protocols. Total RNA samples were stored at -80 °C.

One µg total RNA of each sample was then treated with DNase I (DNA-free™ Kit, Ambion Applied Biosystems, Foster City, CA, USA), in accordance with the manufacturer’s protocol, and reverse transcribed using SuperScript ™ III Reverse Transcriptase (Invitrogen, Carlsbad, CA, USA) and 500 ng oligo(dT)_18_ in reaction volumes of 20 µl, as described in the manufacturer’s protocol. cDNA samples were diluted 1:30 using nuclease-free water (Ambion Applied Biosystems, Foster City, CA, USA), and stored at -20 °C. PCR was carried out using Platinum Taq DNA Polymerase (Invitrogen, Carlsbad, CA, USA), as described by others^40^. PCR products were then ligated into pGEM^®^-T Easy Vector System I (Promega, Madison, WI, USA) and subsequently transformed into CaCl_2_-competent *Escherichia coli* (*E. coli)* cells (TOP10 F; Invitrogen, Carlsbad, CA, USA), and cultured on LB plates containing ampicillin and IPTG/X-gal (Promega, Madison, WI, USA). Positive colonies were checked for inserts of correct size using PCR, and at least eight clones of each gene were cleaned with ExoSAP-IT (Affymetrix, Cleveland, OH, USA) and sequenced (ABI-lab, University of Oslo, Norway), using T7 primers (Invitrogen, Carlsbad, CA, USA). Efforts were made to discover all potential sequences for the genes of interest and variants thereof. Consequently, for each gene, a minimum of seven primer pairs resulted in products that were sequenced. All procedures were carried out in accordance with the manufacturer’s protocol.

Expressed sequence tags (ESTs) obtained from cloning were analysed and paralogs identified. Paralogs are designated with a subscript value. In crucian carp, sequencing analyses revealed three paralogs of the E1α enzyme (PDHE1α_1–3_), two paralogs of the E1β enzyme (PDHE1β_1–2_), E2 enzyme (PDHE2a-b) and the E3 enzyme (PDHE3a-b) and a singleton of E3BP. Partial sequences of genes encoding PDHE1α, PDHE1β and PDHE2 were retrieved from tissues of goldfish and common carp, using the same abovementioned methods. Resulting genes were denoted in accordance with nomenclature given for crucian carp. Additionally, three paralogs were retrieved for ADH8a (ADH8a1–3) in tissues from crucian carp. Partial and full-length sequences were submitted to Genbank (NCBI; www.ncbi.nlm.nih.gov/). Accession numbers are enlisted in Supplementary Table S4.

Full-length sequences of PDHE1α_1_, PDHE1α_3_, PDHE1β_1_, PDHE1β_2_, PDHE2a, PDHE2b, ADH8a1, ADH8a2 and ADH8a3 from crucian carp were obtained using Rapid Amplification of cDNA Ends (RACE), performed on mRNA purified from total RNA using Dynabeads mRNA Direct Kit (Invitrogen, Carlsbad, CA, USA) and using SMART RACE cDNA Amplification kit (Clontech Laboratories Inc., Mountain View, CA, USA), as described by others^41^. GSPs for RACE were designed from sequences obtained from cloning using Primer3, and were synthesized by Invitrogen (Invitrogen, Carlsbad, CA, USA). Primers are enlisted in Supplementary Table S4. RACE PCR was carried out on RACE-ready cDNA using Advantage 2 Polymerase (Clontech Laboratories Inc., Mountain View, CA, USA) and the following hot-start PCR program: 1) 94 °C for 30 sec. 2) 72 °C for 3 min, 3) repeat steps 1–2 4x, 4) 94 °C for 30 s, 5) 70 °C for 30 s 6) 72 °C for 3 min, 7) repeat steps 4–6 4x, 8) 94 °C for 30 s, 9) 68 °C for 30 s, 10) 72 °C for 3 min, 11) 24 repeats of steps 8–10. Cloning and sequencing of all RACE products were carried out as previously described.

Three-dimensional structure models of the crucian carp PDHE1 tetramers of different combinations of paralogs were constructed using Swissmodel (www.swissmodel.expasy.org) and the human crystal structure Protein Databank ID 3EXE^42^; as template. Additionally, a model of the interactions between the E1β dimer and the peripheral subunit binding domain (PSBD) of the E2 monomer was made using the bacterial structure from *Bacillus stearothermophilus* (PDB ID 1W85)^43^. The usability of this template was validated by inspecting the superposition of 3EXE and 1W85 (Supplementary Fig. S10). The resulting model of the interaction surfaces in crucian carp were visualized in PyMOL (The PyMOL Molecular Graphics system, version 1.5.0.4, Schrödinger, LCC), and is presented in Fig. 3A.

A three-dimensional model of ADH8a1 and ADH8a3 from crucian carp was constructed with the crystal structure from Baltic cod (*Gadus morhua callarias;* Protein Databank ID 1CDO)^44^ as template using Swissmodel (www.swissmodel.expasy.org), aiming at elucidating mutations that could result in altered kinetics. The resulting model was visualized in PyMOL (The PyMOL Molecular Graphics system, version 1.5.0.4, Schrödinger, LLC (Supplementary Fig. S9).

### Quantification of gene expression using qPCR

For qPCR, total RNA was extracted from brain, heart, liver, red and white skeletal muscle of crucian carp (anoxia series I), and from normoxic brain and red skeletal muscle of goldfish and common carp, using 15 µl TRIzol/mg, in accordance with the detailed protocol outlined by Ellefsen *et al*.^22^. Prior to homogenization, 20 pg external RNA control gene (*mw2060*) was added per mg tissue. Quality and quantity of the extracted total RNA was assessed using 2100 Bioanalyzer and NanoDrop 2000 UV-Vis Spectrophotometer, as previously described. All samples passed these control tests, and were subsequently DNase I treated and reverse transcribed into cDNA using oligo(dT)_18_ and SuperScript III, as previously described. The final cDNA was eluted 1:30 using nuclease-free water (Life Technologies, Carlsbad, CA, USA) and stored at −20 °C. All procedures were carried out according to the manufacturer’s protocols.

qPCR, using LightCycler480 (Roche Diagnostics, Basel, Switzerland) was performed to assess the abundance of the mRNA of PDHc components in the tissues of the abovementioned species, while mRNA abundance of ADH8a1–3 was quantified in skeletal muscles and liver from crucian carp only. qPCR primers were designed based on the sequences obtained by cloning; when possible, primers were designed to span exon-exon transitions. For each target gene, a minimum of three primer pairs for each gene were tested and their products sequenced. The pair that displayed the highest efficiency, lowest crossing point (Cp) value and most distinct melting curve was adopted. Primers were designed as previously described, and were synthesized by Thermo Fisher Scientific (Waltham, MA, USA). Primers are enlisted in Supplementary Table S4.

qPCR was carried out using LightCycler 480 SYBR Green I Master Kit (Roche Diagnostics, Basel, Switzerland) in a reaction volume of 10 µL, using SYBR Green I Master, primers (100 nM; annealing temperature of 60 °C), cDNA (3 µL 1:30 diluted) and sealed LightCycler® 96 multiwell plates (Roche Diagnostics, Basel, Switzerland). The following qPCR program was used: 1) 95 °C for 10 min, 2) 95 °C for 10 s, 3) 60 °C for 10 s, 4) 72 °C for 13 s 5) repeat steps 2–4 42x. All reactions were carried out in duplicate. In the final analysis of gene expression, the mean values of all qPCR reactions were used. All primers were represented in each plate (for PDHc or ADH, respectively), allowing for subsequent analysis of gene-family profiling^45, 46^. All procedures were carried out according to the manufacturer’s protocol. The relative expression of target genes were calculated from the priming efficiency (E) and the crossing point (Cp) value, and were normalized to the external reference gene *(mw2060)* ^22^. Cp values were calculated for each individual sample using the second derivative maximum method, and were obtained using the LightCycler480 Software (Version 1.5; Roche Diagnostics, Basel, Switzerland). Efficiencies were initially calculated for each individual qPCR reaction using the LinRegPCR software^47^, average priming efficiencies (E_mean_) calculated separately for each primer pair in each tissue were utilized in the final calculations.

### Phylogenetic analyses of PDHc E1 and E2 subunits

Nucleotide sequences from other selected species were gathered from the GenBank (NCBI; www.ncbi.nlm.nih.gov/), The Gene Indices (TGI (formerly TIGR); http://compbio.dfci.harvard.edu/tgi/) and Ensembl Genome Browser (www.ensembl.org/index.html) databases and aligned with the PDHc sequences from crucian carp, goldfish and common carp found in the present study using ClustalX version 2.0.12^39^.

The evolutionary histories of E1α, E1β, and E2 sequences were inferred using both nucleotide sequences and translated amino acid sequences by both Maximum Parsimony and Maximum Likelihood methods in MEGA5.2^48^. In the latter case the best sequence evolution model was determined by using the in-built model selection feature in MEGA5.2. For each subunit, the method generating the most highly resolved tree was chosen. Thus, Supplementary Fig. S7 gives the most parsimonious nucleotide tree for E1α and the tree with the highest log likelihood for both E1β and E2. The percentage of replicate trees in which the associated taxa cluster together in the bootstrap test (1000 replicates) are shown next to the branch points^49^. Accession numbers for sequences included in the analysis are listed in Supplementary Table S11.

### Phylogenetic analysis of ADH8a genes

Amino acid sequences from other selected species were gathered from the GenBank (NCBI; www.ncbi.nlm.nih.gov/), The Gene Indices (TGI (formerly TIGR); http://compbio.dfci.harvard.edu/tgi/) and Ensembl Genome Browser (www.ensembl.org/index.html) databases and aligned with the ADH8a sequences from crucian carp described in the present study using ClustalX version 2.0.12^39^. The evolutionary histories of ADH classes I and III were inferred using the Maximum Likelihood method based on the Jones-Taylor-Thornton (JTT) matrix model^50^ in MEGA5.2^48^, and the resulting tree is shown in Supplementary Fig. S8. A discrete Gamma distribution was used to model evolutionary rate differences among sites (5 categories ( + G, parameter = 1.5325)). The percentage of replicate trees in which the associated taxa cluster together in the bootstrap test (500 replicates) are shown next to the branch points^49^. The phylogenetic analysis was based on 85 amino acid sequences with a total of 302 positions in the final dataset. All positions containing gaps and missing data were eliminated. Accession numbers for sequences included in the analysis are enlisted in Supplementary Table S11.

### Western blot analysis of phosphorylation status of PDHE1α

Frozen crucian carp brain and red skeletal muscle samples from all groups derived from anoxia series I (n = 7 for all groups) were placed in an ice-cold lysis buffer containing 210 mM sucrose, 40 mM NaCl, 30 mM HEPES, 5 mM EDTA, 100 M sodium orthovanadate and 1% Tween-20. Additionally, one tablet complete EDTA-free protease inhibitor (Roche Diagnostics GmbH, Mannheim, Germany) and 250 µL phosphatase inhibitor cocktail 1 were added to 50 mL of extraction buffer (corresponding to 20 mg tissue/mL extraction buffer). Subsequently, the tissue was homogenized using a Polytron PT 1200 homogenizer. Lysates were further centrifuged at 12 000 * g/4 °C/10 min in order to remove any insoluble material. Next, 1% sodium dodecyl sulphate (SDS) was added to the supernatant, and the samples were vortexed for 15 min at room temperature, and later snap-frozen in liquid N_2_ and stored at -80 °C for further analysis.

Protein content in the samples was quantified using Micro BCA protein assay kit (Pierce, Rockford IL). Protein lysates from red muscle (1 µg/lane) and brain (10 µg/lane), respectively, were separated using 10% SDS-PAGE gels and electrophoretically transferred onto a hybond-P membrane (Amersham Biosciences Europe, Freiburg, Germany). To block unspecific binding, membranes were incubated for 2 h in 5% skimmed milk in Tris-buffered saline (20 mM Trizma-base and 140 mM NaCl) with 0.1% Tween-20 (TBST) and subsequently incubated over night with primary antibodies towards either phosphorylated PDHE1α (pSer^293^ (site 1) (AP1062; Merck, Darmstadt, Germany; 1:10000 for red muscle; 1:1000 for brain^51^) or PDHE1α (AV48137; Sigma-Aldrich; 1:1000). The antibody against pSer^293^ (site 1) was chosen as phosphorylation of any of the three phosphorylation sites is sufficient to ablate enzymatic activity of the PDHc^52^, with site 1 being the most frequent target^53, 54^.The epitope of the phospho-Ab was found to be intact in crucian carp. Indeed, the sites of phosphorylation have been shown to be invariant in most vertebrates, supporting the suitability of this antibody for a vast selection of species^51^. After washing with TBST, the membrane was then incubated for 1 h with secondary antibody (goat anti-rabbit; 1:2500, SouthernBiotech, Birmingham, AL, USA), conjugated to horseradish peroxidase. Subsequently, the immunoreactions were visualized by chemiluminescence (ECL + , Amersham Biosciences Europe) and documented using ImageReader LAS-1,000 (Fujifilm Europe). Densitometry of each band was investigated using ImageQuant (Amersham Biosciences Europe). Membranes were stained using Coomassie Brilliant blue (Bio-Rad Laboratories), and scanned (CanonScan Lide 35). Equal loading was investigated using ImageQuant. Membranes displaying uneven blotting were removed from subsequent analyses. The excess E1 transcript level in skeletal muscle was also reflected at the protein level, as Western blot analyses of E1α in lysates from red muscle (Fig. 3A) and brain (Fig. 3B), when diluted to the same degree to avoid saturation, only detected the protein in red muscle (see also Methods and Supplementary Fig. S6 for more details). In contrast, in the anoxia intolerant common carp, overall transcript levels of E1α mRNA were similar between red muscle and brain, with transcription levels being similar to those observed for E1α_1–2_ in *Carassius* brain, and very much below those seen in *Carassius* skeletal muscle (Supplementary Fig. S3).

### Electron microscopy and immunolabeling

Samples for electron microscopy analysis were prepared according to the protocol by Slot and Geuze^55^. In short, a fixed 1 mm^3^ tissue block was infiltrated with 2.3 M sucrose over night at 4 °C and subsequently frozen in liquid nitrogen. Ultrathin cryo-sections (60 nm) were cut in a Reichert Ultracut S microtome equipped with a Leica EMFCS cryo-box and using a Diatome Cryo Immuno knife. Sections were picked up with a droplet containing 2.3 M sucrose and 2% methylcellulose (1:1 mixture), placed on copper grids and stored at 4 °C until further use. For immunolabeling, the grids with sections were washed on droplets of PBS (4 × 5 min), quenched with glycine (0.1% in PBS, 2 × 5 min) and blocked with 1% BSA in PBS (1 × 5 min). Sections were incubated with primary antibody (the same antibodies as were used for the western blot analysis; dilution 1/50 in PBS + 1% BSA) before incubation with 10 nm gold particles conjugated to protein A (PAG; diluted in PBS + 1% BSA). Subsequently, sections were washed in PBS + 0.1% BSA and contrasted with Uranyl acetate by placing the grids on ice on droplets containing a Methyl cellulose-Uranyl Acetate mixture. Three randomized pictures were taken with a Philips CM200 transmission electron microscope from each fish from each exposure group in anoxia series II (N14 °C, N4 °C, A1 and A7). Gold particles were quantified per µm^2^ mitochondria, which in turn had been quantified by a person blinded to the experimental groups. This was performed on three pictures taken randomly from each fish (n = 3 in each treatment). All mitochondria within each picture were quantified (ranging from 3 -11 mitohondria in each).

### Chemicals and reagents

Unless otherwise stated, chemicals and reagents were purchased from Sigma-Aldrich (St. Louis, MO, USA).

### Statistics

For statistical evaluation of qPCR data sets, one-way analysis of variance (ANOVA) with Holm-Sidak post hoc test was performed using SigmaPlot (version 12, Systate Software Inc., San Jose, CA, USA) to compare mRNA expression levels of individual target genes between experimental groups. Data sets illustrating gene-family profiling were arcsine-transformed prior to analysis of mRNA expression levels between experimental groups. All statistical tests were performed separately for each tissue. All data are expressed as means ± standard error of the mean (S.E.M.), unless otherwise stated. Graphs were made using SigmaPlot (version 12, Systate Software Inc., San Jose, CA, USA). Data from Western blots were analysed for statistical variations using GraphPad Prism (version 5; GraphPad Software, La Jolla, CA, USA), and ANOVA with Bonferroni multi comparisons test were carried out on all data sets. Results are presented as means ± standard deviations. Electron microscopy data sets were analysed with ANOVA and Dunnet’s multiple comparison test, and the results are presented as means ± standard deviations. For all statistical tests, the confidence level was set at P < 0.05.

### Data availability

Sequences derived from cloning have been deposited to GenBank, and their accession numbers are enlisted in Supplementary Tables S4 and S11.

## Electronic supplementary material

Supplementary Information
