# Supplementary material for: Extreme anoxia tolerance in crucian carp and goldfish through neofunctionalization of duplicated genes creating a new ethanol-producing pyruvate decarboxylase pathway
Source: Sci Rep. 2017 Aug 11;7:7884. doi: 10.1038/s41598-017-07385-4 (PMC5554223; doi:10.1038/s41598-017-07385-4)
Supplement: Supplementary file 1 — Supplementary Information [file 41598_2017_7385_MOESM1_ESM.docx]

Extreme anoxia tolerance in crucian carp and goldfish through neofunctionalization of duplicated genes creating a new ethanol-producing pyruvate decarboxylase pathway

**Authors:** Cathrine E. Fagernes^1^, Kåre-Olav Stensløkken^2,3^_,_ Åsmund K. Røhr^1^, Michael Berenbrink^4^, Stian Ellefsen^5†§^, Göran E. Nilsson^1*†§^

**Affiliations**

^1^Department of Biosciences, University of Oslo, N-0316 Oslo, Norway.

^2^Institute of Basic Medical Sciences, University of Oslo, N-0372 Oslo, Norway

^3^Center for Heart Failure Research, University of Oslo, N-0317 Oslo. Norway

^4^Institute of Integrative Biology, University of Liverpool, Liverpool L69 7ZB, United Kingdom.

^5^The Lillehammer Research Center for Medicine and Exercise Physiology, Inland Norway University of Applied Sciences, N-2604 Lillehammer, Norway.

*Correspondence to: [g.e.nilsson@ibv.uio.no](mailto:g.e.nilsson@ibv.uio.no)

†Authors contributed equally to this work

§Authors jointly supervised this work

**Supplementary Information**

**
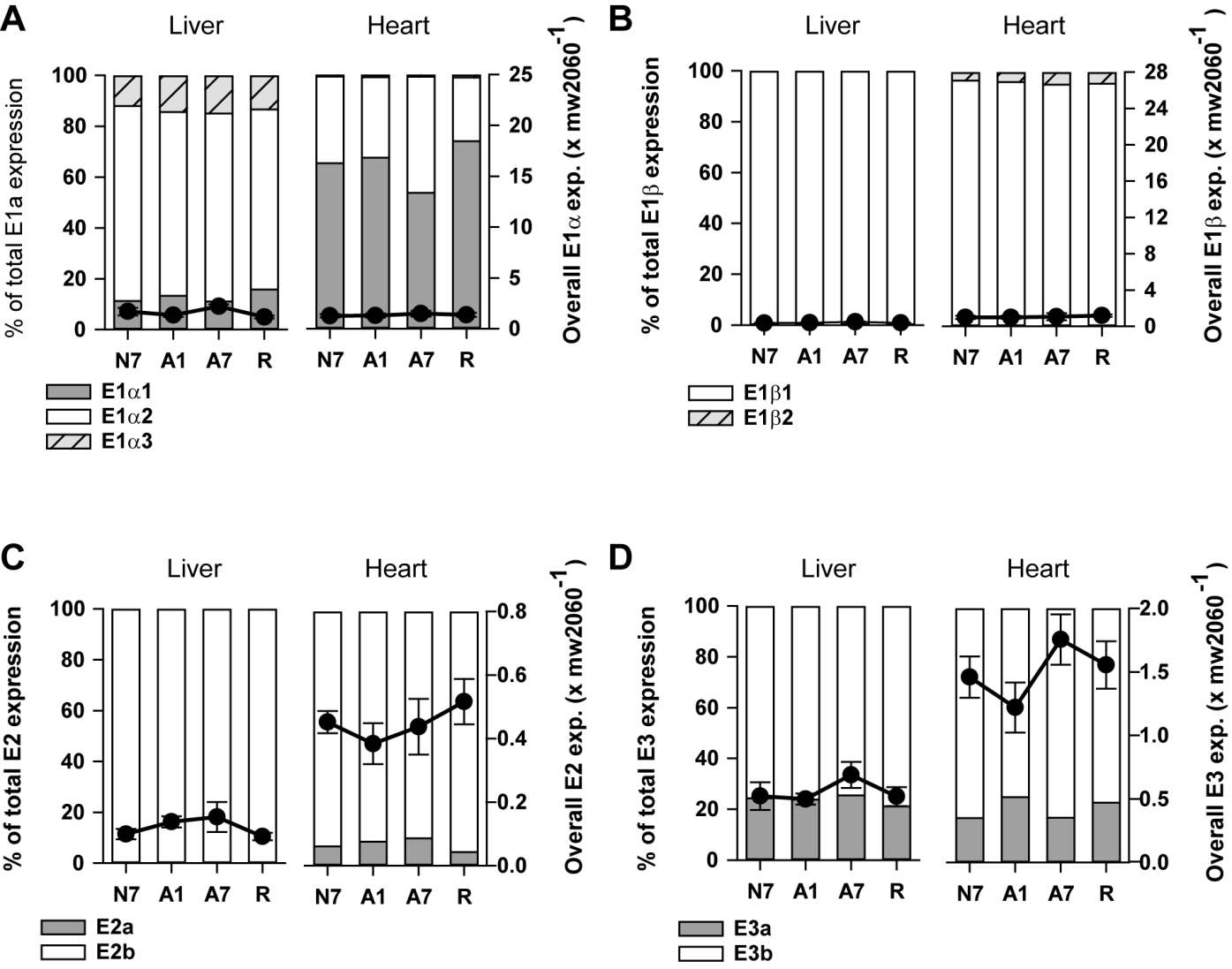
**

Supplementary Figure S1. mRNA transcript levels of PDHc subunits (A) E1α, (B) E1β, (C) E2 and (D) E3 in liver and heart of crucian carp.

X axis show the treatment groups (N7, A1, A7 and R). The left y-axis shows the gene-family profiling of the subunits, illustrating the composition of paralogs within a tissue in the different groups. The right y-axis shows the overall relative within-tissue expression of the subunit in the treatment group in each tissue. Data sets are normalized to the external RNA control mw2060. No statistical differences between groups compared to N7 were found (One-way ANOVA). Values are means ± S.E.M. N = 6-8/group.


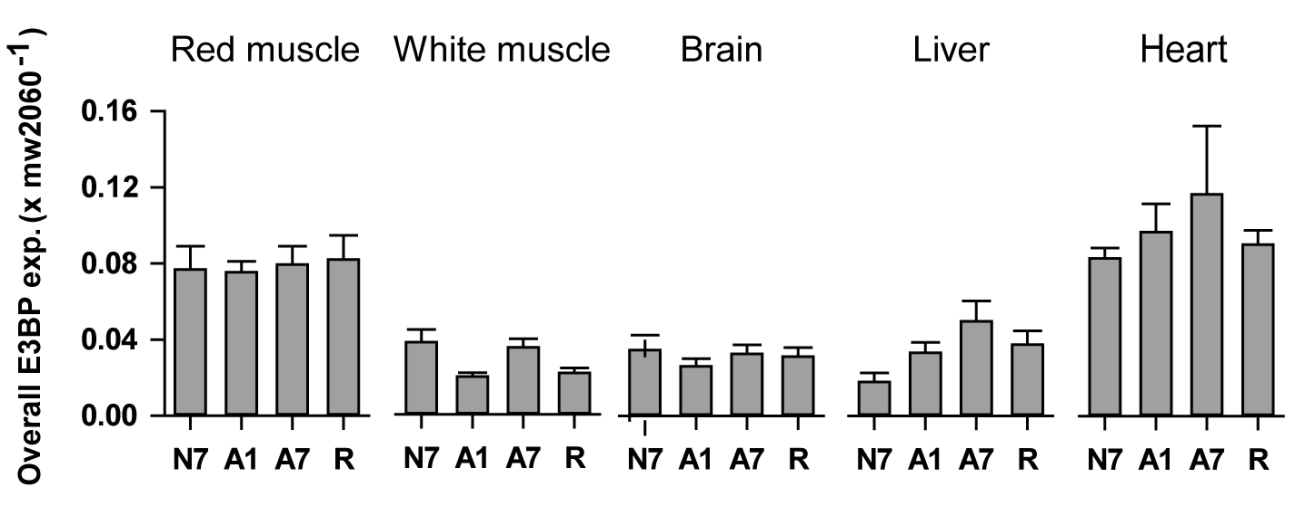


Supplementary Figure S2. mRNA transcript levels of PDHc subunit E3BP red muscle, brain, white muscle, liver and heart of crucian carp.

X axis show the treatment groups (N7, A1, A7 and R). The y-axis shows the relative expression of the subunit in the treatment group in each tissue. Data sets are normalized to the external RNA control mw2060. Values are means ± S.E.M. N = 6-8/group. No statistical differences between groups were found (One-way ANOVA).


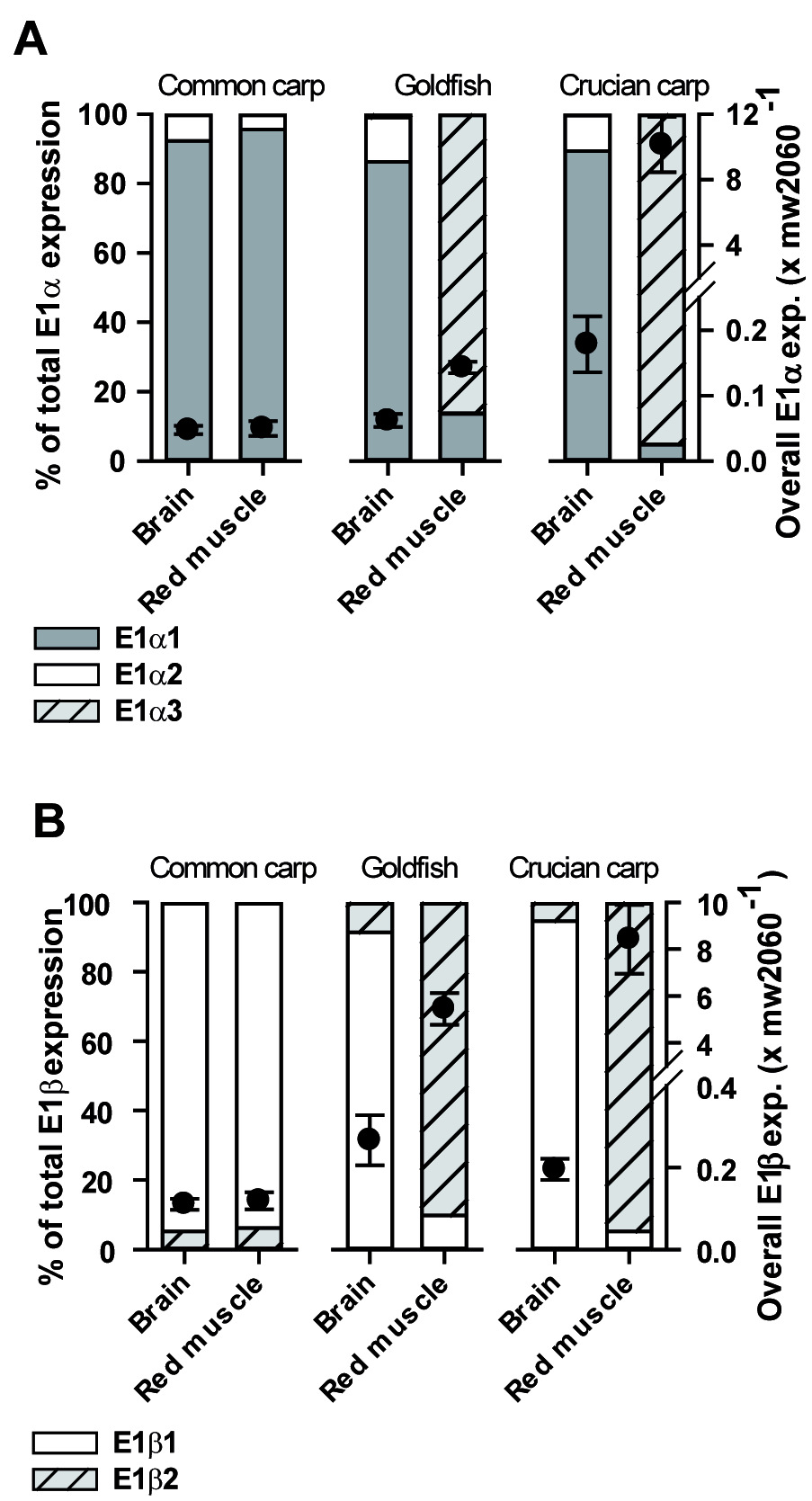


Supplementary Figure S3. mRNA transcript levels of PDHc subunit (A) E1α and (B) E1β in brain and red muscle of common carp, goldfish and crucian carp.

The left y-axes show the gene-family profiling, illustrating the composition of paralogs within a tissue. The right y-axes show the overall relative expression of the subunit in each tissue. Data sets are normalized to the external RNA control mw2060. No statistical difference between brain and red muscle were found in common carp. In goldfish, statistical differences in gene-family profiling between brain and red muscle were found for all paralogs (P<0.001), except E1α_2_ (P>0.05). The overall expression levels of both E1α and E1β were significantly higher in red muscle than brain (P<0.001). In crucian carp, gene-family profiling and overall expression levels of both E1α and E1β were significantly higher in red muscle than brain (P<0.001). Statistical differences not indicated in the figure; One-way ANOVA; Holm-Sidak post-hoc test. N = 4-5 for each tissue.

Supplementary Table S4. Primer sequences

**A**

| **Gene** | **GenBank ID** | **Primers for cloning** | **Primers for RACE** | **Primers for qPCR** |
| --- | --- | --- | --- | --- |
| mw2060 | DQ075244 | - | - | F- GTGCTGACCATCCGAG  R- GCTTGTCCGGTATAACT  E = 1.906 ± 0.001; Cp = 26.1 ± 0.8 |
| E1α_1_ | KF960825 | F– CCCGGACATATGCAGACTTT  R- CCTTCAGCAACGAGATAGGG | 5’– CTCTGTGGAAGCTGCCCTGCATTTT  3’- CACAAAGGACATCCATGCCATCCACTC | F-AGCTGCCTTGCATTTTCGTT  R-GCCTCCCTTACACAAAGGAC  E = 1.915 ± 0.002; Cp = 27.9 ± 0.9 |
| E1α_2_ | KF960847 | F- TATCGCATGATGCAGACCAT  R- TGGTTGCACAAATCTTCCAG | - | F- GGATGGAGCTGCTAATCAGG  R- ACGCTGAGAACATCCATGC  E = 1.927 ± 0.002; Cp = 27.6 ± 0.1 |
| E1α_3_ | KF960826 | F- CCCGGACATATGCAGACTTT  R- CCTTCAGCAACGAGATAGGG | 5’- CTCTGTGGAAGCTGCCCTGCATTTT  3’- CCTTGGTGCCCAGGTATTTGCAGGAA | F- GCACCAAGGATCTGTGTGTC  R- GGATGAATTCGCCTCTCTTG  E= 1.913 ± 0.005; Cp = 26.6 ± 3.5 |
| E1β_1_ | KF960827 | F- CGCATACAAGGTCAGCAGAG  R- CTTCTCCGTTAAAAAGACTCT | 5’- CAGTGACCGTACCAAGCGGCAAAG  3’- TGGCCTCATTTTGGAGTTGGGTCTG | F- CTGTGTCTCGATGCTGCTGT  R- GACCCAACTCCAAAATGAGG  E = 1.915 ± 0.003; Cp = 27.9 ± 0.8 |
| E1β_2_ | KF960828 | F- TCTCCATGCAATCAGTCGAT  R- TGTCCTTGACTTGTGGGATG | 5’- GGTCTTGGCGGCAGAATTGATGATATG  3’- TTCCCACGTGGTCAGTCGGTGTATG | F- GTCACGCTGGTGTCTCATTC  R- CAAAAATCTCTCCCCCGACT  E = 1.884 ± 0.004; Cp = 24.8 ± 3.9 |
| E2a | KF960829 | F- TCTCAAACGGTACCACTCCA  R- GCACCTTTAAACAGGCCAGA | 5’- TCATGTGAGGTGGGTAGGAGCTTCCAG  3’- GCTGCCCCAGCTGTTGCTTCTGT | F- TGGTGGATATCAATGTTGCAGT  R- GCCCAGATTGGAAATAGTGAAG  E = 1.914 ± 0.001; Cp = 28.9 ± 1.6 |
| E2b | KF960830 | F- TCTCAAACGGTACCACTCCA  R- GCACCTTTAAACAGGCCAGA | 5’- AACACACGCCGACCGTCACACAAG  3’- ATCTGCTGCACCCACACCCACAC | F- CACGAGAGGGAAAACTACAACC  R- TCACAGCTCAGAGTCACAGACA  E = 1.914 ± 0.001; Cp = 29.3 ± 0.8 |
| E3a | KF960831 | F- AGCAGCTCAGCTTGGCTTTA  R- ACAGTAGGGTGAGCGTGACA | - | F- CGTCCCTTCACCAGTAACCTC  R- TGGGTCCAGCTACAACATCTC  E = 1.908 ± 0.001; Cp = 29.5 ± 0.7 |
| E3b | KF960848 | F- AGCAGCTCAGCTTGGCTTTA  R- ACAGTAGGGTGAGCGTGACA | - | F- GTCGAGGGTATAGCAGGAGGA  R- ATCCTGTCTGTGTCCTTGTGG  E = 1.908 ± 0.003; Cp = 27.6 ± 0.8 |
| E3BP | KF960844 | F- CTCAGAGCCATCAAACCCAAT  R- TCATCCCTCTGATTCAACAGC | - | F- GAAACCTTGTCAAATGGCTGA  R- ATCTCCACCTGCTTCCAGTCT  E = 1.913 ± 0.003; Cp = 30.7 ± 0.7 |
|  |  |  |  |  |
| mw2060 | DQ075244 | - | - | F- GTGCTGACCATCCGAG  R- GCTTGTCCGGTATAACT  E = 1.888 ± 0.003; Cp = 24.00 ± 1.25 |
| ADH8a1 | JX975106 | F- AAAGCAGCTGGGCAACTAAA  R- CATCGTTGACTTGCTCCAGA | 5’- ACTGGTGCCCATGAACTGCAGGAT  3’- GCCCGGCCAATTCAGCTCATATCT | F- TAGTGTTTTGGTTGGCTGGAC  R- GGTTGACTGCATCATTGACCT  E = 1.900 ± 0.004; Cp = 25.95 ± 0.58 |
| ADH8a2 | JX975105 | F- GGACTTTGTCACACCGACCT  R- ACTCCAGCGCACTTCTCATT | 5’- AGGTCGGTGTGACAAACACCTGTGG  3’- GCTGCGGCATCTCTACTGGATACGG | F- TACCACCCGTTTGAAGTGAAG  R- TCACACAGGTTTGTGTTTGGA  E = 1.857 ± 0.021; Cp = 28.55 ± 0.91 |
| ADH8a3 | JX975104 | F- TGTCACACCGACCTTTACCA  R- CATCGTTGACTTGCTCCAGA | 5’- GCAGAACCTGCATTTTCCACACTGAGAG  3’- GGCTGGACTGATGTGAAGGACTTCTCTG | F- GTGTTTGTCACACCGACCTTT  R- ACCTGCATTTTCCACACTGAG  E = 1.913 ± 0.013; Cp = 19.96 ± 0.91 |

**B**

| **Gene** | **GenBank ID** | **Primers for cloning** | **Primers for qPCR** |
| --- | --- | --- | --- |
| mw2060 | DQ075244 | - | F- GTGCTGACCATCCGAG  R- GCTTGTCCGGTATAACT  E = 1.888 ± 0.001; Cp = 22.3 ± 0.4 |
| E1α_1_ | KF960832 | F- CCCGGACATATGCAGACTTT  R- CCTTCAGCAACGAGATAGGG | F- CATTTGCGAGAATAACAAATACG  R- CATGCCATCCAACCTCAATC  E = 1.883 ± 0.003; Cp = 25.0 ± 0.8 |
| E1α_2_ | KF960823 | F- TATCGCATGATGCAGACCAT  R- TGGTTGCACAAATCTTCCAG | F- GAGATTCAGGAAGTTCGCAGT  R- GGCTCTGGATCAGAGGTAGC  E = 1.921 ± 0.007; Cp = 28.9 ± 0.7 |
| E1α_3_ | KF960824 | F- CCCGGACATATGCAGACTTT  R- CCTTCAGCAACGAGATAGGG | F- GCGTTTTCCTGCAAATACCT  R- GATGCCCCTCTCTCAATAGAA  E = 1.921 ± 0.007; Cp = 26.1 ± 5.7 |
| E1β_1_ | KF960833 | F- CAGTGACCGTACCAAGCGGCAAAG  R- TGGCCTCATTTTGGAGTTGGGTCTG | F- CTGTGTCTCGATGCTGCTGT  R- GACCCAACTCCAAAATGAGG  E = 1.925 ± 0.003; Cp = 23.4 ± 0.7 |
| E1β_2_ | KF960834 | F- GGTCTTGGCGGCAGAATTGATGATATG  R- TTCCCACGTGGTCAGTCGGTGTATG | F- GTCACGCTGGTGTCTCATTC  R- CAAAAATCTCTCCCCCGACT  E = 1.892 ± 0.005; Cp = 24.6 ± 4.9 |

**C**

| **Gene** | **GenBank ID** | **Primers for cloning** | **Primers for qPCR** |
| --- | --- | --- | --- |
| mw2060 | DQ075244 | - | F- GTGCTGACCATCCGAG  R- GCTTGTCCGGTATAACT  E = 1.874 ± 0.002; Cp = 22.6 ± 0.1 |
| E1α_1_ | KF960835 | F- CCCGGACATATGCAGACTTT  R- ATGGAATTGTGGGAGCTCAG | F- CATTTGCGAGAATAACAAATACG  R- CATGCCATCCAACCTCAATC  E = 1.887 ± 0.003; Cp = 26.1 ± 0.1 |
| E1α_2_ | KF960836 | F- TATCGCATGATGCAGACCAT  R- TGGTTGCACAAATCTTCCAG | F- GAGATTCAGGAAGTTCGCAGT  R- GGCTCTGGATCAGAGGTAGC  E = 1.910 ± 0.008; Cp = 29.8 ± 0.4 |
| E1β_1_ | KF960838 | F- GGTCTTGGCGGCAGAATTGATGATATG  R- TTCCCACGTGGTCAGTCGGTGTATG | F- GTCACGCTGGTGTCTCATTC  R- CAAAAATCTCTCCCCCGACT  E = 1.888 ± 0.000; Cp = 25.2 ± 0.1 |
| E1β_2_ | KF960837 | F- CAGTGACCGTACCAAGCGGCAAAG  R- TGGCCTCATTTTGGAGTTGGGTCTG | F- CTGTGTCTCGATGCTGCTGT  R- GACCCAACTCCAAAATGAGG  E = 1.912 ± 0.002; Cp = 29.3 ± 0.0 |

**(A)** Primer sequences for cloning, RACE and qPCR of PDHc and ADH8a in crucian carp.
Primers used to study PDHc gene expression are listed above the black line, while primers for ADH8a studies are enlisted below the line. **(B)** Primers for cloning and qPCR of PDHc in goldfish. **(C)** Primers for cloning and qPCR of PDHc in common carp. F, forward primer; R, reverse primer. Mean priming efficiencies (E) and Crossing point (Cp) values for qPCR primer pairs are given beneath the sequences. Values are means from all groups and tissues ± S.E.M.

Supplementary Table S5. Ratios of PDHc components in tissues of normoxic crucian carp.

|  | E1_Tot_:E2_Tot_ | E1_Tot_:E3_Tot_ | E2_Tot_:E3_Tot_ | EtOH production |
| --- | --- | --- | --- | --- |
| Brain | 4.4 ± 0.6_a_ | 2.5 ± 0.3_ab_ | 0.6 ± 0.1_a_ | No |
| Heart | 5.0 ± 0.5_a_ | 1.6 ± 0.1_a_ | 0.3 ± 0.0_b_ | No |
| Liver | 22.4 ± 2.6_b_ | 4.4 ± 0.8_b_ | 0.2 ± 0.1_b_ | No |
| White muscle | 61.8 ± 4.6_c_ | 58.1 ± 9.0_c_ | 0.9 ± 0.1_a_ | Yes |
| Red muscle | 68.8 ± 6.1_c_ | 61.7 ± 14.4_c_ | 0.8 ± 0.1_a_ | Yes |

Data is based on mRNA transcript levels from N7 groups. Statistical differences between tissues are indicated with dissimilar letters (P<0.050; all data sets were log-transformed prior to analyses. One-Way ANOVA; Holm-Sidak post-hoc test). N = 4-8 for each tissue. Tot = total; EtOH = ethanol).

**
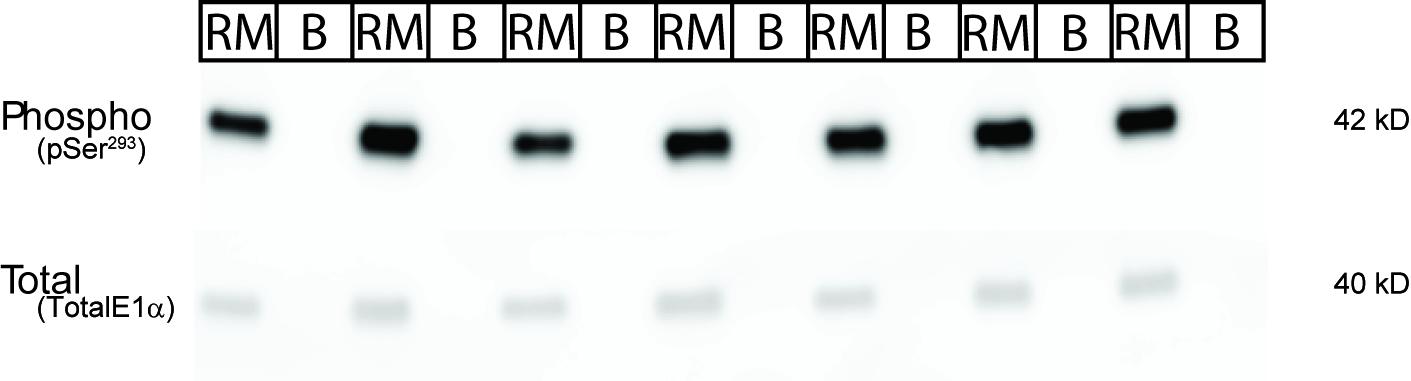
**

**Supplementary Figure S6. Protein expression and phosphorylation of E1α subunits in red muscle (RM) and brain (B) of normoxic crucian carp.**

A comparison of total and phosphorylated protein expression between red muscle and brain was investigated by loading 1 µg of protein lysate per lane to avoid saturation of antibodies in the red muscle samples. Using this concentration, no band was visible for neither the phospho (pSer^293^) nor the total (total E1α) in the brain samples. In the brain blot (Figure 2) both protein concentrations and antibody dilutions were optimized to give visible bands at 40 kD (total E1α) or 42 kD (pSer^293^). We therefore conclude that the protein expression of E1α in the red muscle is much higher than in the brain. kD = kilodalton.


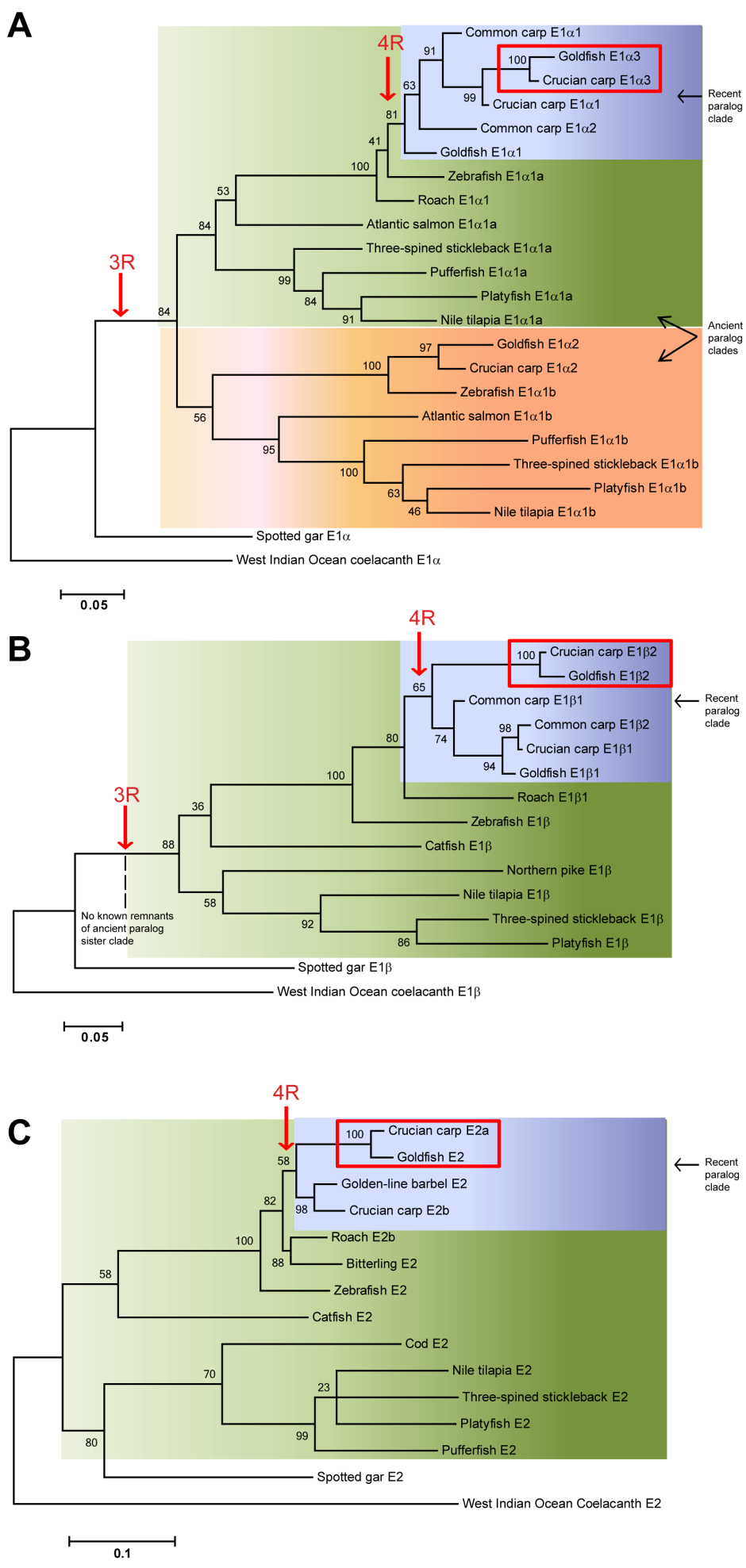


**Supplementary Figure** **S7. PDHc phylogeny.**

Phylogenetic analysis of nucleotide sequences of PDHc subunit E1α **(A)**, E1β **(B)** and E2 (**C**) paralogs. Numbers at each branch point represent bootstrap values from 1000 replicates. All trees are drawn to scale. West Indian Ocean coelacanth was used as outgroup. The teleost wide whole genome duplication (3R) and the cyprinid tetraploidization event (4R) are indicated with red arrows. Accession numbers for sequences used in the analysis are listed in S10A Table. For E1α **(A)**, the most parsimonious tree is shown, based on a total of 832 positions in the final dataset, including 23 nucleotide sequences and with branch lengths calculated using the average pathway method [53] and listed as number of changes over the whole sequence. For E1β **(B)** and E2 (**C**), the maximum likelihood trees are presented with a total of 626 and 592 positions in the final dataset, including 15 and 15 nucleotide sequences, respectively, and with branch lengths measured in the number of substitutions per site. Paralogs dominating the transcript levels in skeletal muscles of ethanol producing species are boxed.


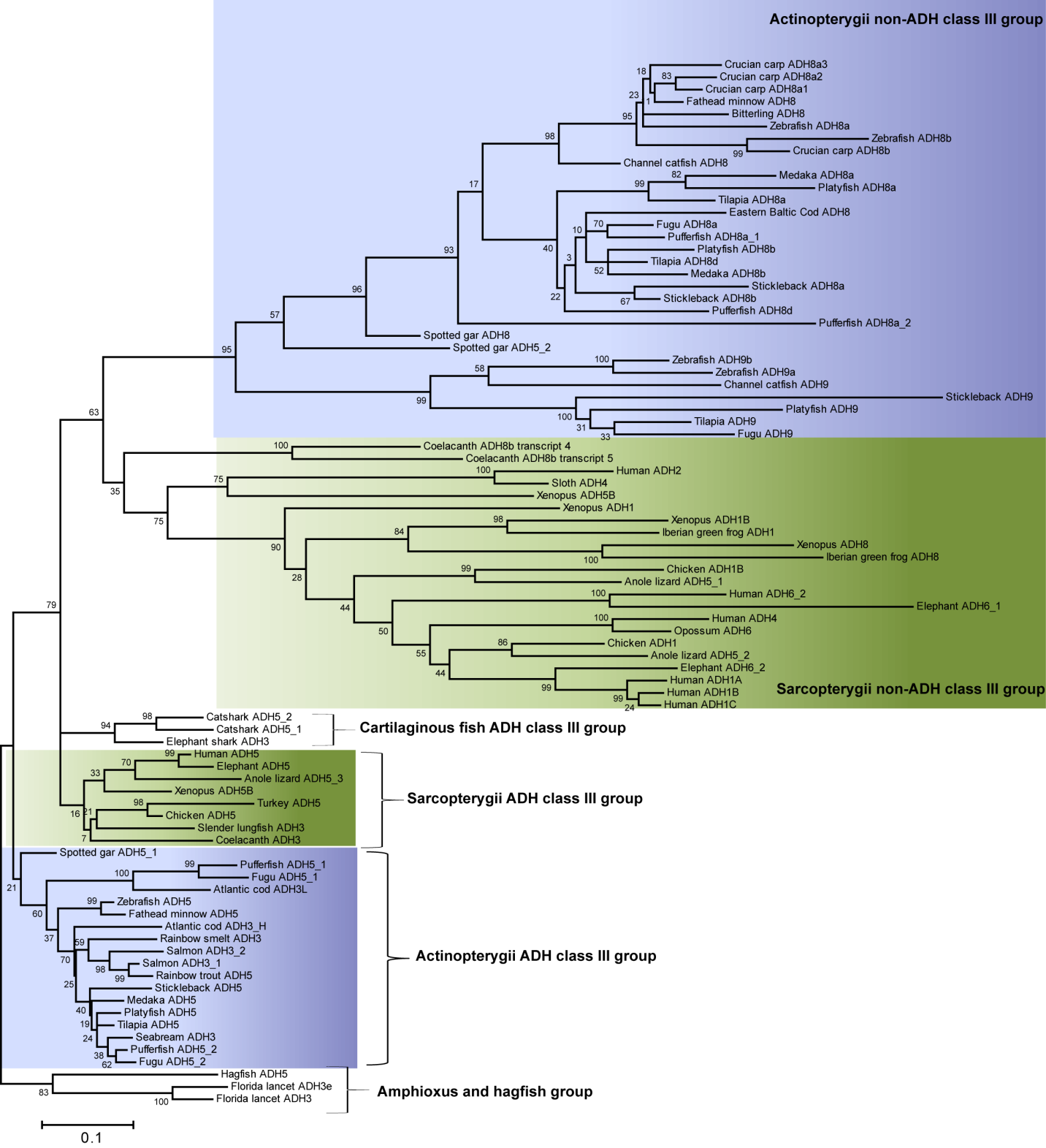
 Supplementary Figure S8. ADH phylogeny.

Maximum likelihood tree of alcohol dehydrogenase (ADH) amino acid sequences. Numbers at each branch point represent bootstrap values from 500 replicates. Tree is drawn to scale, with branch lengths measures in the number of substitutions per site. Accession numbers for sequences used in the analysis are listed in S10B Table.


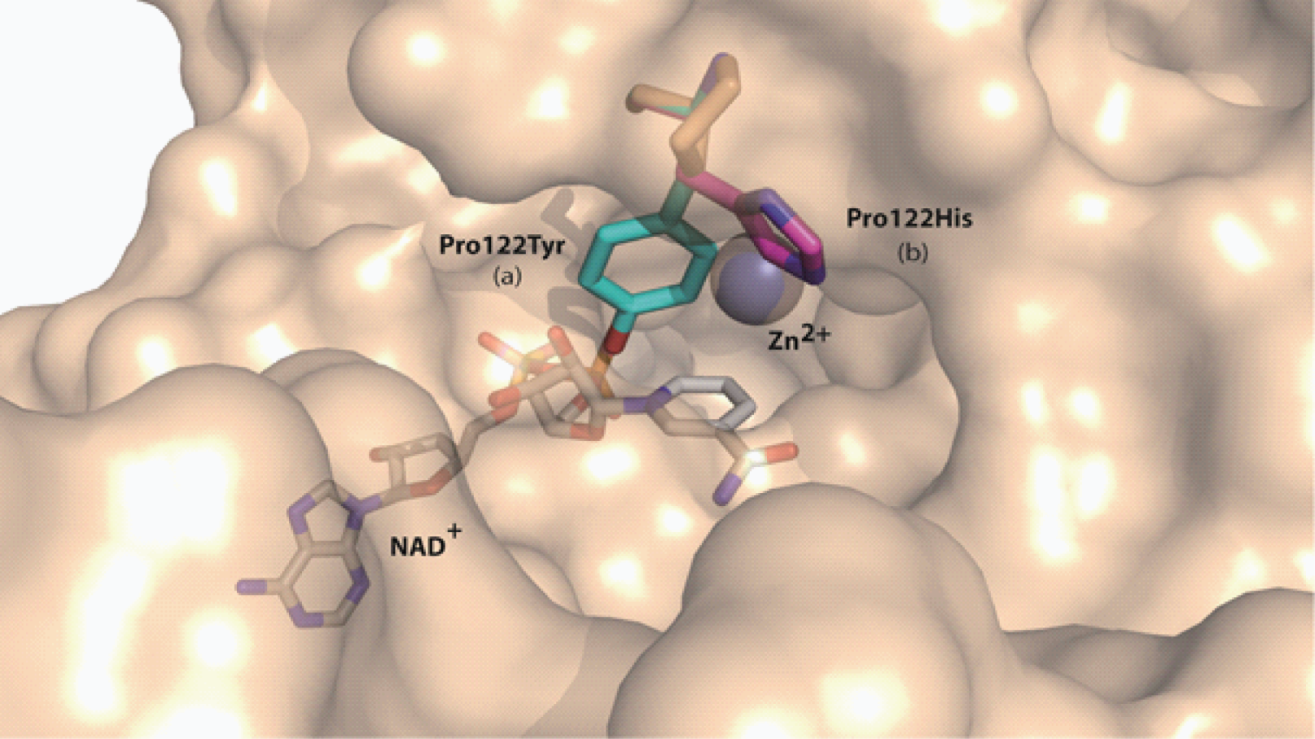


Supplementary Figure S9. *In silico* model of ADH8a1 and ADH8a3.

Three-dimensional structure model of crucian carp ADH8a1 and ADH8a3 modelled with cod ADH8 as template (with cod having Pro in aa 122) indicating the cofactor NAD^+^, Zn^2+^ , and the mutations. The figure shows the substitutions at the entrance point of the substrate channel towards the active site (Pro122Tyr in ADH8a1 (a); Pro122His in ADH8a-3 (b)). None of the modelled mutated amino acid side chains are in contact with the active site cofactors.


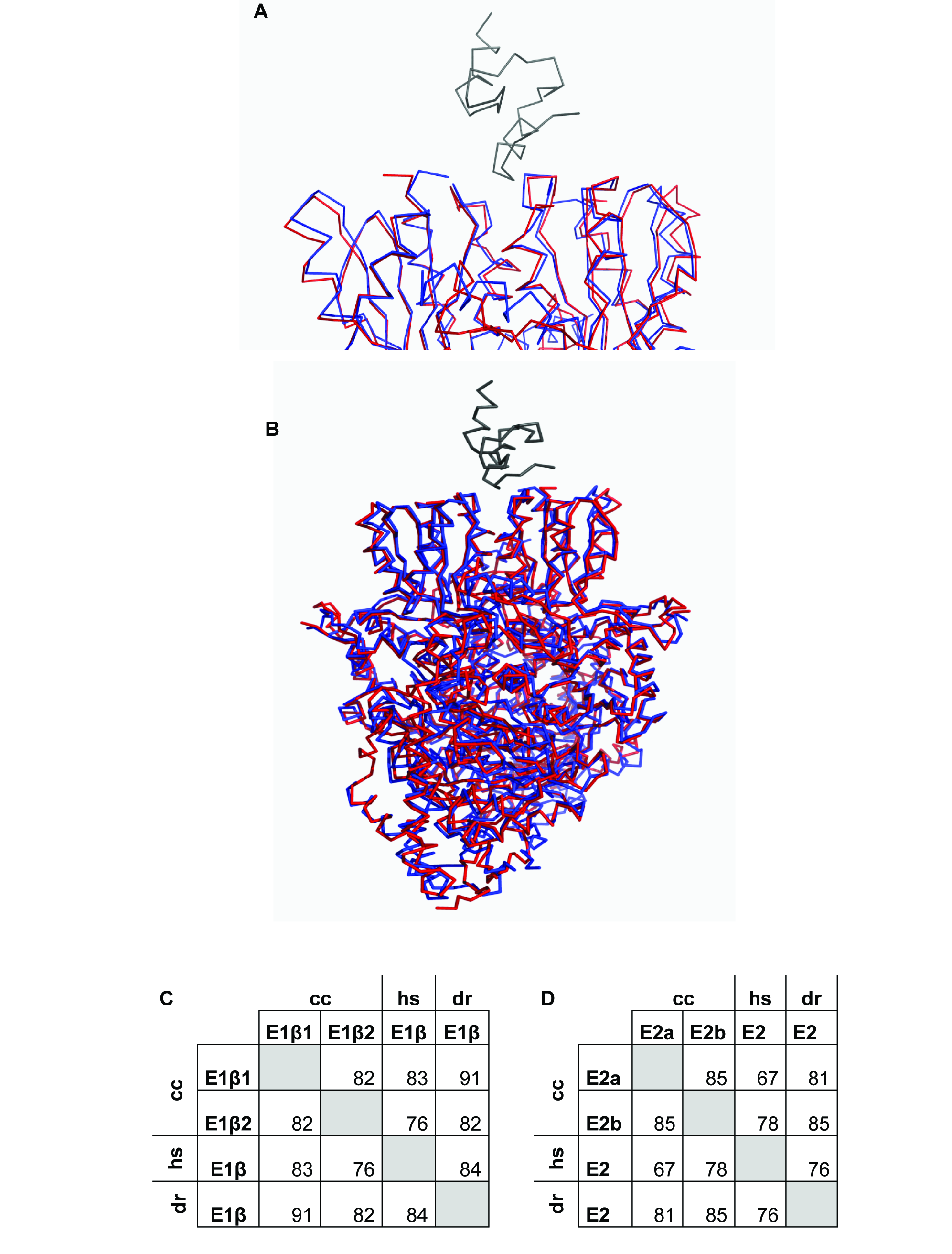


Supplementary Figure S10. Modelling of PDHcE1-E2 complex.

Superposition of PDHc E1-E2 complex from *Bacillus stearathermophilus* (PDB ID 1W85; E1 blue and E2 grey) and human PDHc E1 (red). The Root-mean-squared (RMS) value of the two structures is 1.59 Å, and the high similarity of the folds indicates that the two structures combined are good templates for building the crucian carp E1-E2 complex model. A) PSBD of E2 monomer and the E2-binding sites of the E1β dimer; B) the E1 tetramer and the PSBD of the E2 monomer; C) Identities (in percent) of the E1β protein sequences of crucian carp (cc), human (hs) and zebrafish (dr); D) Identities (in percent) of the E2 protein sequences of crucian carp (cc), human (hs) and zebrafish (dr).

Supplementary Table S11. Accession numbers for phylogenetic analysis PDH and ADH

**A**

| Species | Gene | GenBankID | Ensembl ID | TGI ID |
| --- | --- | --- | --- | --- |
| Goldfish  *(Carassius auratus)* | E1α_1_  E1α_2_  E1α_3_  E1β_1_  E1β_2_ | KF960832  KF960823  KF960824  KF960833  KF960834 |  |  |
|  | E2 | KF960849 |  |  |
| Crucian carp  *(Carassius carassius)* | E1α_1_  E1α_2_  E1α_3_  E1β_1_  E1β_2_  E2a  E2b | KF960825  KF960847  KF960826  KF960827  KF960828  KF960829  KF960830 |  |  |
| Zebrafish  *(Danio rerio)* | E1α1a  E1α1b  E1β  E2 | NM213393  NM213154 | ENSDART00000123299  ENSDART00000016502 |  |
| Common carp  *(Cyprinus carpio)* | E1α_1_  E1α_2_  E1β_1_  E1β_2_ | KF960835  KF960836  KF960838  KF960837 |  |  |
| Atlantic salmon  *(Salmo salar)* | E1α1a  E1α1b | BT046034 |  | TC168839 |
| Three-spined stickleback  *(Gasterosteus aculeatus)* | E1α1a  E1α1b  E1β  E2 |  | ENSGACT00000027396  ENSGACT00000016953  ENSGACT00000005219  ENSGACT00000027361 |  |
| Platyfish  *(Xiphophorus maculatus)* | E1α1a  E1α1b  E1β  E2 |  | ENSXMAT00000013083  ENSXMAT00000011821  ENSXMAT00000008858  ENSXMAT00000011017 |  |
| Roach  *(Rutilus rutilus)* | E1α1  E1β1 | KF960841  KF960843 |  |  |
|  | E2 | KF960845 |  |  |
| Bitterling  *(Rhodeus amarus)* | E2 |  |  |  |
| Pufferfish  *(Takifugu rubripes)* | E1α1a  E1α1b  E2 | CAAB02013035 | ENSTRUT00000043475  ENSTRUT00000043813 |  |
| Nile Tilapia  *(Oreochromis niloticus)* | E1α1a  E1α1b  E1β  E2 | XM003447658 | ENSONIT00000006466  ENSONIT00000010357  ENSONIT00000006236 |  |
| West Indian Ocean Coelacanth  *(Latimeria chalumnae)* | E1α  E1β  E2 | AFYH01087568 | ENSLACT00000012327  ENSLACT00000017910 |  |
| Spotted gar  *(Lepisosteus oculatus)* | E1α  E1β  E2 | AHAT01003223  AHAT01029316 | ENSLOCT00000001733 |  |
| Catfish  *(Ictalurus punctatus)* | E1β  E2 | NM001201084 |  | TC59073 |
| Northern pike  *(Esox lucius)* | E1β | BT079237 |  |  |
| Atlantic cod  *(Gadus morhua)* | E2 |  | ENSGMOT00000002995 |  |
| Golden-line barbel  *(Sinocyclocheilus angustiporus)* | E2 | GAHO01091414 |  |  |

**B**

| Species | Gene | GenBankID | Ensembl ID | TGI ID |
| --- | --- | --- | --- | --- |
| Crucian carp  *(Carassius carassius)* | ADH8a1  ADH8a2  ADH8a3  ADH8b | JX975106  JX975105  JX975104  JX975102 |  |  |
| Iberian green frog  *(Phelophylax perezi)* | ADH1  ADH8 | P22797  O57380 |  |  |
| Bitterling  *(Rhodeus amarus)* | ADH8 | KJ126794 |  |  |
| Zebrafish  *(Danio rerio)* | ADH5  ADH8a  ADH8b  ADH9a  ADH9b | NP571924  AAK97852  AY309074  NP991204  NP956749 |  |  |
| Human  *(Homo sapiens)* | ADH1A  ADH1B  ADH1C  ADH2  ADH4  ADH5  ADH6_2 | P07327  P00325  P00326  AAH22319  P40394  AAH14665  NP000663 |  |  |
| Fathead minnow  *(Pimephales promelas)* | ADH5  ADH8 |  |  | TC49822  TC48035 |
| Channel catfish  *(Ictalurus punctatus)* | ADH8  ADH9 |  |  | TC76033  TC54937 |
| Eastern Baltic cod  *(Gadus morhua callarias)* | ADH8 | P26325 |  |  |
| Atlantic hagfish  *(Myxine glutinosa)* | ADH5 | P80360 |  |  |
| Medaka  (Oryzias latipes) | ADH5  ADH8a  ADH8b |  | ENSORLP00000006535  ENSORLP00000024580  ENSORLP00000022083 |  |
| Platyfish  *(Xiphophorus maculatus)* | ADH5  ADH8a  ADH8b  ADH9 |  | ENSXMAP00000002332  ENSXMAP00000015353  ENSXMAP00000015382  ENSXMAP00000002315 |  |
| Tilapia  *(Oreochromis niloticus)* | ADH5  ADH8a  ADH8d  ADH9 |  | ENSONIP00000001368  ENSONIP00000024531  ENSONIP00000024534  ENSONIP00000001377 |  |
| Fugu  *(Takifugu rubripes)* | ADH5_1  ADH5_2  ADH8a  ADH9 |  | ENSTRUP00000020384  ENSTRUP00000039680  ENSTRUP00000006693  ENSTRUP00000039767 |  |
| Pufferfish  *(Tetraodon nigroviridis)* | ADH5_1  ADH5_2  ADH8a_1  ADH8a_2 |  | ENSTNIP00000002273  ENSTNIP00000016001  ENSTNIP00000003265  ENSTNIP00000008345 |  |
| Stickleback  *(Gasterosteus aculeatus)* | ADH5  ADH8a  ADH8b  ADH9 |  | ENSGACP00000021750  ENSGACP00000007245  ENSGACP00000021758  ENSGACP00000007192 |  |
| Spotted gar  *(Lepiosteus oculatus)* | ADH5_1  ADH5_2  ADH8 |  | ENSLOCP00000015035  ENSLOCP00000015044  ENSLOCP00000014545 |  |
| Coelacanth  *(Latimeria chalumnae)* | ADH3  ADH8b.4  ADH8b.5 | AAS49517 | ENSLACP00000022184  ENSLACP00000002561 |  |
| Three-toed sloth  *(Choloepus hoffmanni)* | ADH4 |  | ENSCHOP00000007922 |  |
| Western clawed frog  *(Xenopus tropicalis)* | ADH1  ADH1B  ADH5A  ADH5B  ADH8 |  | ENSXETP00000020503  ENSXETP00000020507  ENSXETP00000020485  ENSXETP00000063992  ENSXETP00000059494 |  |
| Chicken  *(Gallus gallus)* | ADH1  ADH1B  ADH5 |  | ENSGALP00000019979  ENSGALP00000031486  ENSGALP00000019979 |  |
| Anole lizard  *(Anolis carolinensis)* | ADH5_1  ADH5_2  ADH5_3 |  | ENSACAP00000011468  ENSACAP00000011892  ENSACAP00000014133 |  |
| Elephant  *(Loxodonta africana)* | ADH5  ADH6_1  ADH6_2 |  | ENSLAFP00000000841  ENSLAFP00000011030  ENSLAFP00000018189 |  |
| Opossum  *(Monodelphis domestica)* | ADH6 |  | ENSMODP00000025907 |  |
| Florida lancet  *(Branchiostoma floridae)* | ADH3_1  ADH3e | AAK26852  AAF73255 |  |  |
| Seabream  (Sparus aurata) | ADH3 | AAB41888 |  |  |
| Rainbow trout  *(Oncorhynchus mykiss)* | ADH5 | FP320537 |  |  |
| Atlantic salmon  *(Salmo salar)* | ADH3_1  ADH3_2 | ACN62250  ACN10409 |  |  |
| Rainbow smelt  *(Osmerus mordax)* | ADH3 | ACO09512 |  |  |
| Atlantic cod  *(Gadus morhua)* | ADH3H  ADH3L | P81600  P81601 |  |  |
| Slender lungfish  *(Protopterus dolloi)* | ADH3 | AAS49516 |  |  |
| Turkey  *(Meleagris gallopavo)* | ADH5 |  | ENSMGAP00000011695 |  |
| Elephant shark  *(Callorhinchus milii)* | ADH3 | AFM90649 |  |  |
| Catshark  *(Scyliorhinus canicula)* | ADH5_1  ADH5_2 | AAS49606  P86884 |  |  |

Accession numbers for nucleotide sequences included in the phylogenetic analysis of PDH E1α, E1β and E2 evolution (A) and accession numbers for amino acid sequences included in the phylogenetic analysis of ADH evolution (B).
